# Perilipin-2 modulates dietary fat-induced microbial global gene expression profiles in the mouse intestine

**DOI:** 10.1186/s40168-017-0327-x

**Published:** 2017-09-06

**Authors:** Xuejian Xiong, Elise S. Bales, Diana Ir, Charles E. Robertson, James L. McManaman, Daniel N. Frank, John Parkinson

**Affiliations:** 1 Molecular Medicine, Hospital for Sick Children, 686 Bay Street, Toronto, M5G 0A4 ON Canada; 2 Division of Reproductive Sciences, University of Colorado, 12700 E. 19th Avenue, Aurora, 80045 CO USA; 3 Division of Infectious Diseases, University of Colorado, 12700 E. 19th Avenue, Aurora, 80045 CO USA; 4 Microbiome Research Consortium, University of Colorado, 12700 E. 19th Avenue, Aurora, 80045 CO USA; 5 The Center for Human Nutrition, University of Colorado, 12700 E. 19th Avenue, Aurora, 80045 CO USA; 6 Department of Molecular Genetics, University of Toronto, 1 King’s College Circle, Toronto, M5S 1A8 ON Canada; 7 Department of Biochemistry, University of Toronto, 1 King’s College Circle, Toronto, M5S 1A8 ON Canada

**Keywords:** Perilipin-2, Microbiome, Metatranscriptomics, Metabolic pathways, Lipid uptake

## Abstract

**Background:**

Intestinal microbiota are critical determinants of obesity and metabolic disease risk. In previous work, we showed that deletion of the cytoplasmic lipid droplet (CLD) protein perilipin-2 (Plin2) modulates gut microbial community structure and abrogates long-term deleterious effects of a high-fat (HF) diet in mice. However, the impact of Plin2 on microbiome function is unknown.

**Results:**

Here, we used metatranscriptomics to identify differences in microbiome transcript expression in WT and Plin2-null mice following acute exposure to high-fat/low-carbohydrate (HF) or low-fat/high-carbohydrate (LF) diets. Consistent with previous studies, dietary changes resulted in significant taxonomic shifts. Unexpectedly, when fed a HF diet, the microbiota of Plin2-null and WT mice exhibited dramatic shifts in transcript expression despite no discernible shift in community structure. For Plin2-null mice, these changes included the coordinated upregulation of metabolic enzymes directing flux towards the production of growth metabolites such as fatty acids, nucleotides, and amino acids. In contrast, the LF diet did not appear to induce the same dramatic changes in transcript or pathway expression between the two genotypes.

**Conclusions:**

Our data shows that a host genotype can modulate microbiome function without impacting community structure and identify Plin2 as a specific host determinant of diet effects on microbial function. Along with uncovering potential mechanisms for integrating how diet modulates host and microbial metabolism, our findings demonstrate the limits of 16S rRNA surveys to inform on community functional activities and the need to prioritize metatranscriptomic studies to gain more meaningful insights into microbiome function.

**Electronic supplementary material:**

The online version of this article (10.1186/s40168-017-0327-x) contains supplementary material, which is available to authorized users.

## Background

Obesity is a substantial public health concern with increasing prevalence worldwide [1]. Since 1980, worldwide obesity has more than doubled and in 2014, 13% of adults were considered obese [2]. A critical determinant of obesity risk is the intestinal microbiota [3–5], which has been linked to a wide range of co-morbidities, including metabolic syndrome [4], gastrointestinal disease [6], type-2 diabetes (T2D), [7] and non-alcoholic fatty liver disease (NAFLD) [8, 9]. Metabolic functions encoded by the intestinal microbiome have a significant impact on the host [10]. In addition to the extraction of key nutrients such as amino acids and vitamins, fermentation by the intestinal microbiota produces short-chain fatty acids (SCFAs), such as butyrate, which are the primary energy sources for colonic epithelia and essential to the development of villus morphology within the GI tract [11, 12]. Although previous studies have shown that diet can have a dramatic impact on the composition and function of the gut microbiome [10, 13, 14], much less is known concerning the role of the host in shaping diet-microbiome interrelationships.

Cytoplasmic lipid droplets (CLDs) are intracellular organelle-like structures that play critical regulatory roles in lipid homeostasis [15–21]. Unlike other lipoprotein particles that enter circulation (e.g., LDL, HDL), CLDs form exclusively intracellular, cytoplasmic organelles storing neutral lipids [15–20]. Within enterocytes, CLDs are thought to function as sites of temporary storage for dietary lipids, which eventually are incorporated into chylomicrons and secreted into the lymphatic capillaries draining the villi of the small intestines [15, 22]. Underlying the formation and regulation of CLDs are members of the perilipin family which act as protein scaffolds [23–26]. Within this family, perilipin-2 (*Plin2*) is among the most highly expressed, particularly in the liver and small intestine [21, 27–31]. In a previous work, we documented in a transgenic whole-body *Plin2* knockout mouse model (Plin2-null) that Plin2 modulates rapid (< 4 days) effects of diet on fecal lipid levels, enterocyte CLD contents, and fuel utilization properties of mice that correlate with differences in their gut microbial communities [32]. What is not known is how interactions between diet and Plin2 genotype modify the *functional*, as opposed to compositional, properties of the gut microbiome.

To date, most microbiome investigations have relied on the use of 16S rRNA surveys. Although such surveys provide details of community structure, they provide only limited functional insights. Algorithms such as PICRUSt [33] can be applied to deduce the functional capacity of a microbiome based on taxonomic abundances; however, such capacity does not directly translate to functional activity. Consequently, whole microbiome RNASeq (metatranscriptomics) has emerged as a powerful technology to interrogate microbiome function and define members of microbial communities in terms of their functional activities [34]. Here, we apply metatranscriptomics to compare the impact of two dietary regimes (high-fat/low-carbohydrate vs. low-fat/high-carbohydrate) on the structure and function of the intestinal microbiomes of Plin2-null and WT mice. Note that throughout, we define community structure on the basis of expressed transcripts rather than, for example, marker genes such as 16S rDNA genes.

## Results

### Knockout of Plin-2 has minimal impact on the biodiversity of the mouse intestinal microbiome in the context of low-fat and high-fat diets

In previous work, we used 16S rRNA surveys and metagenomics to show that both dietary fat content and Plin2-null genotype could significantly and independently impact gut microbiome composition, diversity, and function [32]. To derive a more mechanistic understanding of the relationship between dietary fat content, *Plin2* genotype, and microbiome function, we undertook a metatranscriptiomics analysis of the colon contents of both wild type (WT) and Plin2-null (Plin2) mice shifted from chow to either a low-fat/high-carbohydrate (LF) or high-fat/low-carbohydrate (HF) diet for 4 days. Bulk RNA was prepared from colon contents and sequenced, resulting in ~ 419 million sequence reads from 16 mice (4 replicates of each diet/genotype combination) of which ~ 234 million reads (55.8%) were of putative bacterial mRNA origin (Table 1; see Additional file 1: Table S1 for additional information on sequence reads).

**Table 1:** Summary of sequence read processing

Genotype	Diet	Total reads	Putative mRNA reads	% putative mRNA	Annotated mRNA reads	% of putative mRNA reads annotated	Unique transcripts^a^	Unique enzymes^b^
Plin2KO	HF	23,259,351	12,202,390	52.46	8,324,287	68.22	354,623	1467
Plin2KO	HF	26,835,502	14,899,443	55.52	12,247,229	82.20	421,650	1499
Plin2KO	HF	30,521,645	16,599,182	54.38	13,388,961	80.66	403,966	1464
Plin2KO	HF	26,597,134	14,650,846	55.08	11,925,997	81.40	373,227	1465
Plin2KO	LF	22,294,860	12,361,287	55.44	10,610,304	85.83	331,728	1421
Plin2KO	LF	24,177,170	13,568,281	56.12	11,565,876	85.24	367,713	1453
Plin2KO	LF	27,308,384	15,414,942	56.45	13,153,267	85.33	337,142	1467
Plin2KO	LF	30,451,443	17,236,702	56.60	14,227,281	82.54	361,329	1481
WT1	HF	21,588,643	11,895,187	55.10	8,943,569	75.19	321,451	1462
WT2	HF	18,204,894	9,395,383	51.61	7,349,179	78.22	303,773	1453
WT3	HF	25,596,552	14,678,726	57.35	12,343,681	84.09	349,102	1466
WT4	HF	26,696,428	15,035,608	56.32	11,779,646	78.34	361,607	1468
WT5	LF	26,214,964	14,929,947	56.95	12,324,432	82.55	333,978	1480
WT6	LF	25,606,683	15,475,773	60.44	14,246,619	92.06	210,470	1407
WT7	LF	31,198,548	17,212,742	55.17	13,361,302	77.62	359,230	1471
WT8	LF	32,317,247	18,081,900	55.95	14,541,413	80.42	380,172	1474

*HF* high fat, *LF* low fat

^a^Defined as distinct transcripts identified in each sample

^b^Defined as distinct enzyme classification (EC) numbers

**Fig. 1:**
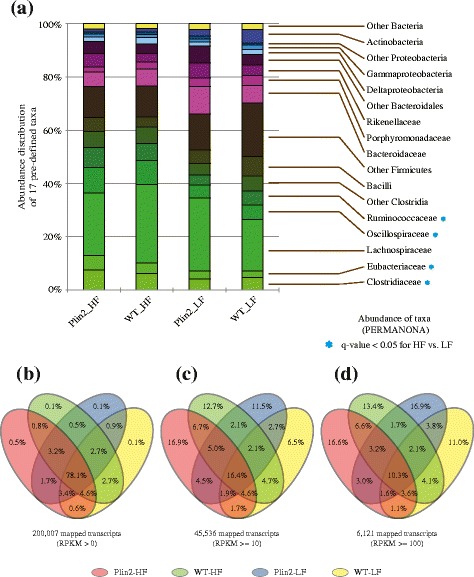
Abundance distribution of microbiome across four mice samples. **a** Abundance distribution of 17 predefined bacterial taxa for all four mouse genotype/diet groups (Plin2-HF, WT-HF, Plin2-LF, and WT-LF). Blue asterisks indicate the significant shifts in taxon abundance in comparisons of both Plin2 and WT mice fed different diets (*p* value < 0.05). **b**–**d** Venn diagrams of number of mapped microbial transcripts for four samples, Plin2-HF (*red*), WT-HF (*green*), Plin2-LF (*blue*), and WT-LF (*yellow*) with different cutoffs: **b** RPKM > 0, **c** RPKM ≥ 10, and **d** RPKM ≥ 100

Applying our previously developed metratranscriptome analysis pipeline [35], reads were filtered and subsequently assembled into contigs (see “Methods”). Contigs and unassembled reads were then passed through a tiered set of sequence similarity searches using BWA, BLAT, and DIAMOND [36–38] against databases of microbial genomes and bacterial non-redundant proteins, resulting in taxonomic and functional annotations for each contig/read (see Additional file 1: Table S1 for detailed statistics of assembly and annotation). A total of 200,007 distinct bacterial transcripts were identified across all samples (Additional file 2: Table S2). With the exception of Fisher’s Alpha between Plin2-HF and Plin2-LF samples, no significant difference was observed in measures of alpha or beta diversity across the four sample types (Table 2). To investigate changes in specific taxonomic groups, initial assignments were placed into one of 17 pre-defined taxa that encompass most mammalian gut bacterial diversity (Fig. 1a). Consistent with our previous study [32], reads associated with Firmicutes and, to a lesser extent, Bacteroidetes dominated each sample, with *Lachnospiraceae* (of the phylum Firmicutes) being the most abundant family. As before, we also found that the Plin2 mice which shifted to a LF diet (Plin2-LF) exhibited a lower incidence of reads assigned to Firmicutes relative to other samples. Principal components analysis (PCA) revealed no significant separation between four pairwise comparisons (Plin2-HF vs. Plin2-LF; Plin2-HF vs. WT-HF; Plin2-LF vs. WT-LF; and WT-HF vs. WT-LF—non-parametric multiple analysis of variance (PERMANOVA); Additional file 3: Figure S1A). In addition, PERMANOVA tests revealed no significant differences in taxonomic abundance when we compare the two genotypes as a function of diet, or conversely when we compare the two diets as a function of genotype. Furthermore, when we focused on specific taxonomic groups, PERMANOVA tests identified significant differences in read abundance only when comparing HF and LF diets independent of genotype (i.e., combining WT and Plin2 samples); specifically, of the 17 pre-defined taxa, *Clostridiaceae*, *Eubacteriaceae*, *Oscillospiraceae*, and *Ruminococcaceae* display significantly elevated levels of gene expression under a HF diet (Fig. 1).

**Table 2:** Biodiversity analysis across four mice samples. Presented are five indices of alpha diversity and one for beta diversity

Sample	Species richness	Chao 1	F-alpha^a^	Shannon	Simpson	Beta diversity^b^
Plin2-HF	1517 ± 2.9	1561 ± 1.3	134.7 ± 2.66	4.56 ± 0.04	0.97 ± 0.002	0.96 ± 0.002
WT-HF	1513 ± 9.8	1557 ± 0.35	136.0 ± 2.62	4.55 ± 0.08	0.97 ± 0.004	0.96 ± 0.006
Plin2-LF	1492 ± 20.2	1563 ± 4.18	131.3 ± 0.58	4.61 ± 0.16	0.98 ± 0.004	0.94 ± 0.013
WT-LF	1512 ± 26.4	1570 ± 0.25	131.7 ± 3.23	4.56 ± 0.36	0.97 ± 0.023	0.96 ± 0.017

Values given are means with standard deviations

^a^Applying the two sample *t* test for the four types of sample comparisons, only the comparison of Fisher’s Alpha between Plin2-HF and Plin2-LF samples revealed a statistically significant difference in diversity (*p* < 0.05)

^b^Beta diversity was calculated as the number of species in a sample/total_number of species across all 16 samples

In summary, these broad taxonomic analyses indicate that across the four sample types (diet x genotype), only the dietary regime, and not the *Plin2* genotype, had a significant impact on microbiome composition at the phylum/family level. These results corroborate our previous 16S rRNA-based analyses in which *Plin2* genotype had minimal impact on microbiota composition in animals fed either HF or LF diets.

### Diet and Plin2 deletion alter microbiome gene expression

We next examined if genotype influenced the functional distribution of reads (Fig. 1b–d). Of the 200,007 unique microbial transcripts identified across all samples, a core set of 156,289 transcripts (78.1%) were identified in all four diet/genotype groups (i.e., Plin2-HF, Plin2-LF, WT-HF, WT-LF). Of the remaining 43,718 transcripts absent in at least one of the four samples, only a small proportion of transcripts were uniquely associated with either *Plin2* genotype or diet (Fig. 1b). Interestingly, samples from the same genotype (i.e., Plin2-HF and Plin2-LF; WT-HF and WT-LF) shared a greater proportion of expressed transcripts (1.7 and 2.7%, respectively) than samples sharing diets (0.8% for Plin2-HF and WT-HF, and 0.9% for Plin2-LF and WT-LF), suggesting that *Plin2* genotype was a factor in modifying transcript profiles between treatment groups. When only transcripts with at least moderate or high levels of expression (defined as ≥ 10 and ≥ 100 reads per kilobase of transcript per million mapped reads (RPKM), respectively) in at least one sample type were considered, the proportion of shared, core transcripts dropped to 16.4 and 10.3%, respectively, with many transcripts displaying elevated expression only in a single sample type (Fig. 1c, d). For example, across the 6121 highly expressed transcripts (≥ 100 RPKM), 16.6% were specific to Plin2-HF samples, 13.4% were specific to WT-HF samples, 16.9% were specific to Plin2-LF samples, and 11% were specific to WT-LF samples. Given that their high expression is limited to specific samples, such transcripts might reflect key responses to changes in environmental conditions imposed by different diets and/or genotypes.

To further examine the impact of *Plin2* genotype and diet on gene expression, we used DESeq [39] to identify 3777 bacterial transcripts that exhibited significant differences in expression across the four sample types (Additional file 4: Table S3). PCA based on the expression of these transcripts revealed a significant separation between Plin2 and WT mice fed a HF diet (*q* value < 0.05, PERMANOVA). Significant differences were also observed in HF vs. LF dietary regimes fed to Plin2 mice (*q* value < 0.05, PERMANOVA), indicating altered community transcriptional activity (Additional file 3: Figure S1B). Hence, through metatranscriptomics, PCA revealed that although the taxonomic distribution of the gut microbiota was unaffected by host *Plin2* genotype, the microbiota of Plin2 and WT mice exhibited distinct gene expression profiles when fed a HF diet.

**Fig. 2:**
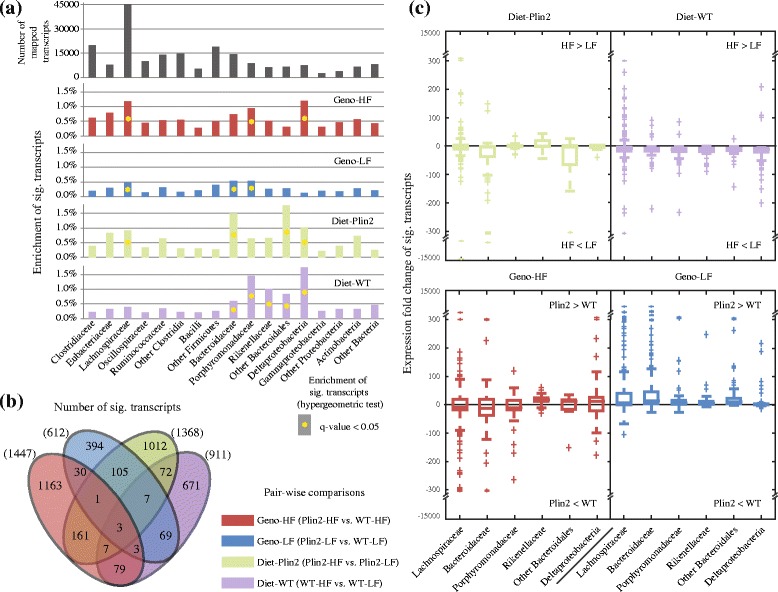
Microbial transcripts expressed differentially across taxa for different sample-wise comparisons. **a** Bar charts, from top to bottom, show the total number of mapped microbial transcripts and the enrichment of significant differentially expressed transcripts (significant transcripts) identified from each of four pairwise comparisons (see inset key) across 17 predefined taxa. Detailed *q* values are provided in Additional file 5: Table S4. **b** Venn diagram of number of significant transcripts for all four pairwise comparisons. **c** Boxplots indicating distribution of expression fold change (*eFC*) of significant transcripts from each pairwise comparison. Only the eight taxonomic groups enriched in significant transcripts are shown

We next explored whether the 3777 differentially expressed transcripts were significantly enriched in any of the 17 pre-defined, gut-dominant bacterial taxa. Of the 200,007 unique transcripts identified in our analysis, the three largest represented taxa were *Lachnospiraceae*, *Clostridiaceae*, and “Other Firmicutes” (Fig. 2a, top panel and Additional file 5: Table S4). Transcripts exhibiting significant differential expression were identified in 6 of the 17 pre-defined taxa, including both abundant (e.g., *Lachnospiraceae*) and rarer taxonomic groups (Fig. 2a). For example, the increased abundance of *Lachnospiraceae* reads and enrichment in differentially expressed transcripts in three of four comparisons (i.e., Plin2 vs. WT on HF diet, Plin2 vs. WT on LF diet, and Plin2 on HF vs. LF diet; Geno-HF, Geno-LF, and Diet-Plin2 panels, respectively, in Fig. 2a) is consistent with our previous 16S-based study, which suggested an increased abundance of this taxon, albeit without statistical significance, under a HF diet for both Plin2 and WT mice [32]. Notably, we did not identify differential expression in this taxon between WT mice fed LF and HF diets, suggesting that loss of *Plin2* function alters the expression of *Lachnospiraceae* genes, irrespective of dietary factors (Diet-WT panel, Fig. 2a). Furthermore, *Deltaproteobacteria* was enriched in differentially expressed transcripts in comparisons between Plin2 and WT animals raised on a HF diet, in addition to dietary (HF vs. LF) comparisons for both Plin2 and WT mice. *Porphyromonadaceae* was also enriched in differentially expressed transcripts in comparisons between Plin2 and WT mice fed HF diets, in addition to comparisons of the two genotypes fed a LF diet and the two diets fed to WT mice.

Contrasting significantly differentially expressed transcripts across the four sample-wise genotype/diet comparisons (Fig. 2b) revealed that most significant differentially expressed transcripts were unique to each group. The WT vs. Plin2 comparison under a LF diet exhibited the fewest significant transcripts (612 total, 394 specific to this comparison), whereas the WT vs. Plin2 comparison under a HF diet exhibited the most (1447 total, 1163 specific to this comparison). Three transcripts were identified in all four comparisons (i.e., Plin2 vs. WT on HF vs. LF diet): a SSU ribosomal protein S3P (EC1_07050) putatively expressed by *Eubacterium cylindroides* T2–87; an environmental response regulator, CLS_10500, putatively expressed by *Clostridium cf. saccharolyticum*, a member of the *Lachnospiraceae*; and a flagellin (WP_031391632.1) putatively expressed by *Clostridium sp. KNHs209*. CLS_10500 encodes a CheY-like receiver domain and a winged-helix DNA-binding domain. Together, these two elements form part of a modular OmpR-like two-component signaling system [40] which has been shown in *Escherichia coli* to have roles in osmoregulation [41], chemotaxis [42], and the sensing and transport of nutrients, including carbohydrates [43] and fatty acids [44]. Under a LF diet, CLS_10500 exhibited almost 90-fold higher expression in Plin2 mice relative to WT (95.6 ± 112.6 vs. 1.10 ± 1.32 RPKM for Plin2 vs. WT; Additional file 4: Table S3), but was diminished over 200-fold (1.49 ± 1.96 vs. 345.2 ± 685.7 RPKM for Plin2 vs. WT) under a HF-diet. The WP_031391632.1 transcript is also of interest since in some organisms, and flagellin is regulated by the OmpR system [45]. Here, we found that WP_031391632.1 was again significantly upregulated in Plin2-null mice relative to WT under a LF diet, but was significantly downregulated in Plin2-null mice under a HF diet (RPKM = 124.9, 1.7, 0.4, and 47.9 for Plin2-LF, Plin2-HF, WT-LF, and WT-HF, respectively). These findings illustrate the interlinked effects of *Plin2* and diet on bacterial transcript expression. Of note, previous studies have reported that flagellins secreted by motile bacteria are important determinants of host-microbiome interactions and gut homeostasis [46–48]. Further analyses of the 417 transcripts representing flagellin from different taxa found 37 transcripts that exhibited significant differential expression, although no clear pattern of direction of change in expression was observed (Additional file 4: Table S3).

Because of the association of specific taxa with specific differentially expressed transcripts, we were interested in examining the magnitude and direction of change of all transcripts, broken down by a taxonomic group (Fig. 2c). Focusing on the six taxa we previously identified as being enriched in these transcripts, for the dietary comparisons (i.e., Plin2-HF vs. Plin2-LF and WT-HF vs. WT-LF), only modest changes in expression were observed, with most taxa demonstrating an overall decrease in expression under the HF diet (Fig. 2c, top panels). For the genotype comparisons, we found that three of the six taxa (*Rikenellaceae*, other *Bacteroidales*, and *Deltaproteobacteria*) exhibited a general increase in transcript expression in Plin2-null mice relative to WT under a HF diet. Furthermore, under a LF diet, differentially expressed transcripts from all six taxa were upregulated in Plin2 mice relative to WT. Together, these findings suggest that, unlike taxonomic composition, Plin2 genotype and not diet, had a greater impact on the magnitude of change in transcript expression.

Among the transcripts that were significantly differentially expressed in the HF comparison (Plin2-HF vs. WT-HF), 44 displayed fold-changes greater than 300; most of them derived from *Lachnospiraceae*, *Bacteroidaceae*, and *“*other Firmicutes” (Additional file 2: Table S2). These transcripts included CK5_14390, a flavoprotein from *Ruminococcus obeum*, which was highly expressed only in the Plin2-HF samples (RPKM = 2412 ± 2340; compared to < 2 for the other three sample types). Flavoproteins are involved in butyrate synthesis pathways, a key metabolite for maintaining gut homeostasis and epithelial integrity [49]. Conversely, RHOM_00695, a UDP-4-dehydro-6-deoxy-2-acetamido-d-glucose 4-reductase from *Roseburia hominis*, displayed considerably reduced expression only in the Plin2-HF samples (RPKM = 29.7 ± 35.5; compared to > 2000 for the other three sample types). RHOM_00695 belongs to the short-chain dehydrogenase/reductase family and is essential for *Helicobacter pylori* pathogenesis, with roles in the biosynthesis of flagella and lipopolysaccharide [50–52].

### Plin2 genotype and dietary fat/carbohydrate balance modulate microbial functional pathways operating in the gut

**Fig. 3:**
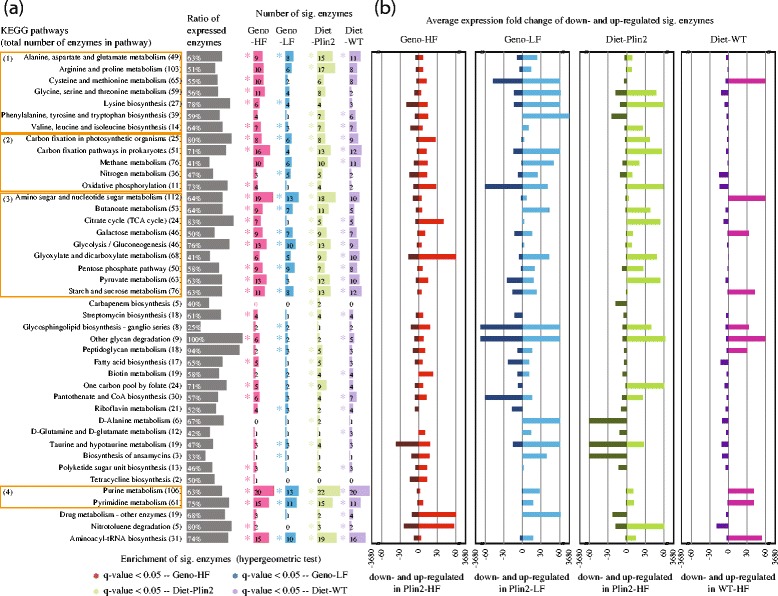
Distribution of enzymes in metabolic pathways for different sample-wise comparisons. For each of the 36 KEGG metabolic pathways listed, the number (**a**) and average expression fold change (**b**) of significantly differentially expressed enzymes (significant enzymes) are indicated for each of the four pairwise comparisons. Detailed *q* values are provided in Additional file 6: Table S5. Significant enzymes are defined as enzymes to which at least one significantly differentially expressed transcript is mapped (see “Methods”). Numbers in brackets after pathway name indicate the total number of enzymes associated with each pathway. Twenty-one of the 36 pathways belong to one of the four KEGG-defined super families: (1) amino acid metabolism, (2) energy metabolism, (3) carbohydrate metabolism, and (4) nucleotide metabolism. The ratio of expressed enzymes indicates the proportion of enzymes associated with that pathway that was expressed in at least one of the samples. Asterisks indicate pathways that were statistically significantly enriched in significant enzymes (*p* value < 0.05) for each genotype/diet comparison, e.g., Geno-HF (*red*), Geno-LF (*blue*), Diet-Plin2 (*green*), and Diet-WT (*purple*)

To further explore the functional consequences of changes in microbial community gene expression profiles, differentially expressed transcripts were mapped to metabolic enzymes and subsequently grouped into pathways as defined by the Kyoto Encyclopedia of Genes and Genomes (KEGG; [53]). Gene set enrichment analysis identified 42 of 144 pathways that were enriched in at least one of the four pairwise comparisons of genotype/diet groups (Fig. 3 and Additional file 6: Table S5). Twenty-three of these pathways were associated with the production and/or degradation of amino acids, energy, carbohydrates, or nucleotides (Fig. 3a). Although we note considerable overlap in enriched pathways in the pairwise comparisons of the genotype/diet groups, the comparison of Plin2-null vs. WT mice fed a LF diet exhibited the fewest number of enriched pathways (20 pathways). Consistent with the taxonomic analysis of differentially expressed transcripts (Fig. 2a), the greatest abundances of differentially expressed enzymes were observed in the comparisons of (1) Plin2-null vs. WT mice fed a HF diet (153 enzymes in 27 pathways) and (2) Plin2-null mice fed HF vs. LF diets (164 enzymes in 26 pathways).

Analyses of expression fold-change revealed that WT mice fed LF and HF diets exhibited the least change in microbial pathway expression, with differentially expressed genes in 25 of 42 pathways exhibiting < 10 fold-change (Fig. 3a). In comparison, microbiota of Plin2 mice exhibited a global upregulation of most enriched pathways under a HF diet with 24 of 42 pathways exhibiting > 10-fold change, suggesting that the microbiota of Plin2-null mice either were more sensitive than WT to increased dietary fat or that *Plin2* deletion altered the physiological and/or nutritive landscape of the gut. This is further shown in the comparison of Plin2 and WT mice fed a HF diet (Fig. 3b), in which differentially expressed enzymes tended to be upregulated in Plin2-null relative to WT animals. Perhaps reflecting the relatively low number of differentially expressed enzymes, comparisons between Plin2-null and WT mice fed a low-fat/high-carbohydrate diet exhibited more extreme distributions of fold change of expression.

PCA analysis of differentially expressed enzymes revealed significant differences in clustering between the Plin2-null and WT mice fed a HF diet, as well as WT mice fed HF and LF diets (*q* value < 0.05 for both; Additional file 3: Figure S1D). However, only the former exhibited significant differences in PCA clusters at the level of the pathway (Additional file 3: Figure S1E). Together, these results reveal that differential expression of microbial transcripts between Plin2 and WT mice extends to changes in the expression of multiple enzymes having the potential to alter metabolic activity in the microbiome.

### A high-fat diet in the context of Plin2 knockout results in coordinated patterns of expression among consecutive enzymes involved in energy metabolism

**Fig. 4:**
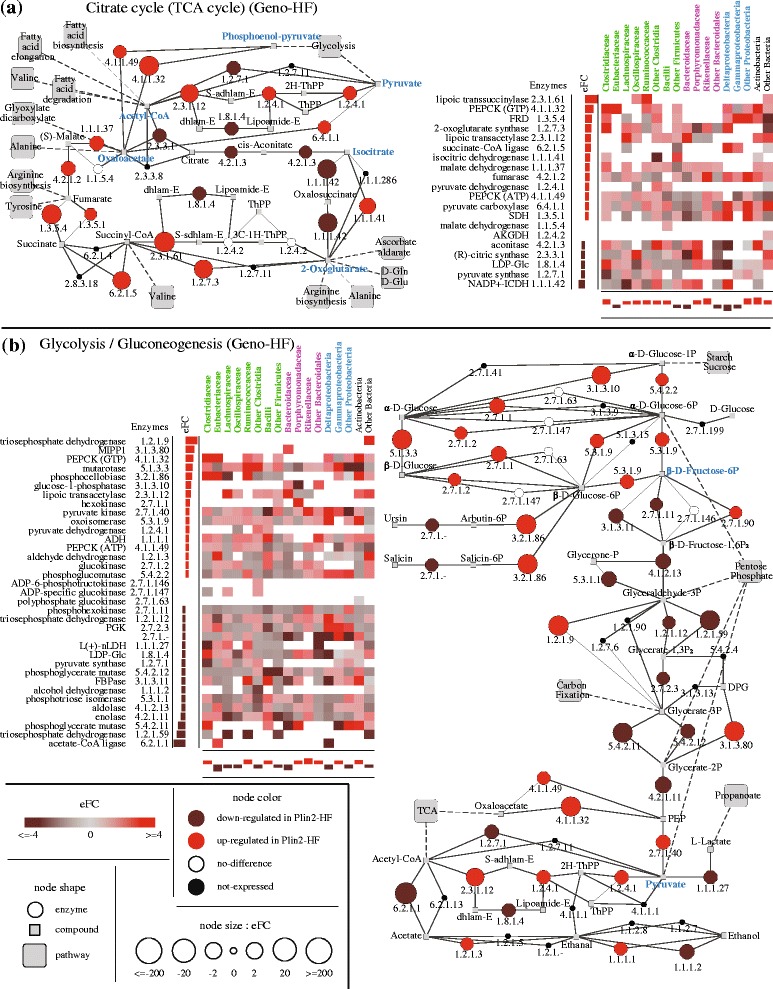
Comparison of enzyme expression between Plin2-HF and WT-HF mice. Two pathways are shown: the TCA cycle (**a**) and glycolysis and gluconeogenesis metabolic pathway (**b**). For each pathway, circular nodes indicate enzymes, with size indicating a relative difference in expression between sample types (Plin2-HF vs. WT-HF) and color indicating the direction of change (see inset key). Associated heatmaps indicate global changes in expression for each enzyme, in addition to taxon-specific changes in expression for each of the 17 defined taxa colored according to phylum. Key metabolites mentioned in the text are indicated with bold blue text. The following abbreviations are used: S-adhlam-E (S-acetyldihydrolipoamide-E), 2H–ThPP (2-hydroxyethyl-ThPP), dhlam-E (dihydrolipoamide-E), S-sdhlam-E (S-succinyldihydrolipoamide-E), and 3C-1H-ThPP (3-carboxy-1-hydroxypropyl-ThPP)

In the previous section, we identified several metabolic pathways exhibiting differential expression across genotype/diet groups. Of particular interest were comparisons between Plin2-null and WT mice fed a HF diet, given the role of Plin2 in lipid homeostasis. Although both groups exhibited similar taxonomic distributions, 33 KEGG-defined pathways were enriched in differentially expressed genes in either WT or Plin2 groups on HF diet (Fig. 3a). Here, we further explore changes in expression in several pathways involved in energy metabolism and the production of co-factors, amino acids, and butyrate (Fig. 4 and Additional file 7: Figure S2, Additional file 8: Figure S3, Additional file 9: Figure S4, Additional file 10: Figure S5, Additional file 11: Figure S6, Additional file 12: Figure S7).

Focusing on energy metabolism, most of the enzymes involved in the TCA cycle (i.e., including those not identified through DESeq as differentially expressed transcripts) were upregulated in Plin2 mice (Fig. 4a and Additional file 13: Table S6). All of these enzymes, except isocitrate dehydrogenase (EC:1.1.1.41), catalyze consecutive reactions linking the glycolytic metabolites, pyruvate and phosphoenolpyruvate (PEP), to key intermediates used in the synthesis of fatty acids and amino acids, as well as the utilization of reducing equivalents [54]. Such “coordinated” patterns of expression may result in potential metabolic channeling that connect both acetyl-CoA, a key intermediate in fatty acid metabolism (through pyruvate), and PEP to oxaloacetate and subsequently to 2-oxoglutarate (α-ketoglutarate) and the synthesis of amino acids, through NADH-dependent fumarate reduction, a part of the bacterial redox system [54].

Analysis of the microbial taxa in the context of this pathway revealed several taxa with relatively consistent upregulation of the enzymes that may drive channeling of PEP and/or Acetyl CoA, with at least 6 of the 12 enzymes involved displaying some level of upregulation (Fig. 4a inset heatmap). These include both taxa displaying an overall upregulation of TCA cycle enzymes (C*lostridiaceae*, *Actinobacteria*, “other Proteobacteria,” and “other Clostrida”) and those displaying an overall downregulation of TCA cycle enzymes (*Deltaproteobacteria* and “other Firmicutes”). In contrast, the three enzymes: citric synthase (EC:2.3.3.1); aconitase (EC:4.2.1.3), and NADP-dependent isocitrate dehydrogenase EC:1.1.1.42), linking acetyl-CoA to 2-oxoglutarate through isocitrate were downregulated in Plin2 compared with WT. Such downregulation was possibly driven by changes in the expression of these three enzymes in *Lachnospiraceae*, *Deltaproteobacteria*, and “other Bacteroidiales.” Thus, although similar taxonomic profiles were found in WT and Plin2 mice, notably different taxa exhibited divergent responses in TCA cycle regulation, which presumably reflect inter-taxa metabolic optimizations related to microbial and host responses to HF diet. For example, *Bacteroidaceae* downregulated the expression of six enzymes and upregulated the expression of five enzymes in the TCA cycle in Plin2 compared with WT mice.

Among the other three pairwise comparisons of diet/genotype groups (i.e., Geno-LF, Diet-Plin2, and Diet-WT), coordinated patterns of differential expression across consecutive enzymes were also observed in both the Geno-LF comparison and the Diet-WT comparison (Additional file 7: Figure S2). In the former, enzymes linking succinate to isocitrate and acetyl-CoA via oxaloacetate are upregulated in Plin2 mice, while in the latter enzymes linking citrate to fumarate via 2-oxaglutarate are consistently downregulated in WT mice fed a HF diet.

Interestingly, analysis of the glycolysis pathway also revealed consistent differential regulation of enzymes performing consecutive reactions (Fig. 4b). For example, enzymes involved in the production of PEP from β-d-fructose-6-phosphate were largely downregulated in Plin2 compared with WT animals on HF diet, whereas those acting directly on various forms of glucose (e.g., phosphoglucomutase—EC:5.4.2.2 and glucose-1-phosphatase—EC:3.1.3.10) were upregulated. As with the TCA cycle, specific taxa yielded distinct patterns of regulation across glycolysis, with 8 of the 17 taxa displaying an overall regulation of this pathway and the other 9 displaying an overall downregulation of this pathway. Focusing on the ten enzymes linking β-d-fructose-6-phosphate to PEP, we observe the greatest contributions to the coordinated pattern of downregulation from Lachnospiracea (8 of 10 enzymes downregulated), “other Firmicutes” (7 enzymes), “other Bacteroidales” (7 enzymes), Bacteroidaceae (6 enzymes), and “other bacteria” (6 enzymes). Other taxa display more heterogeneous patterns of expression; for example, *Clostridiaceae*, *Eubacteriaceae*, and *Bacilli* respectively feature four, five, and four downregulated enzymes and six, three, and five upregulated enzymes.

For the other diet-genotype comparisons, although other parts of the pathway displayed heterogeneous responses to diet and *Plin2* genotype, enzymes involved in the production of pyruvate from β-fructose-6-phosphate were largely downregulated under a HF diet in both WT and Plin2-null animals (Additional file 8: Figure S3).

Next, with important roles in the TCA cycle and fatty acid metabolism, we examined the pantothenate and CoA biosynthetic pathway (Additional file 9: Figure S4 and Additional file 10: Figure S5). As before, we note consistent patterns of regulation involving consecutive reactions. For example, enzymes linking valine and uracil were consistently upregulated in Plin2-null mice compared to WT mice fed a HF diet, while those linking pantothenate to CoA were largely downregulated (i.e., upregulated in WT mice). Interestingly, this latter pattern was also observed in WT mice fed a HF diet compared to those fed a LF diet. This again highlights the coordinated regulation of consecutive enzymes in these pathways with the potential to redirect metabolic flux between key metabolites. Furthermore, taxon-specific patterns of expression were observed; enzymes in the pathway associated with *Rikenellaceae*, *Deltaproteobacteria*, *Clostridiaceae*, *Eubacteriaceae*, and *Baciilli* were largely upregulated in the Plin2-mice compared to WT mice fed a HF diet while other groups displayed more mixed patterns of expression.

Finally, focusing on butyrate metabolism (Additional file 11: Figure S6 and Additional file 12: Figure S7), we again identify patterns of elevated expression of consecutive enzymes in Plin2 mice relative to WT mice fed either a high- or low-fat diet that may help channel metabolites. In particular, we note the upregulation of many enzymes linking acetyl-CoA with butyrate for both dietary comparisons and fumarate to acetyl CoA for the HF diet comparison. Interestingly, we note little correlation in taxonomic responses to the two dietary comparisons. For example for Plin2-null mice, five taxa (*Lachnospiraceae*, *Ruminococcaceae*, “other Clostridia,” *Bacteroidaceae*, and *Porphyromonadaceae*) exhibit an overall downregulation of the pathway under a HF diet relative to WT but an overall upregulation of the pathway under a LF diet.

Furthermore, we note that two enzymes that help link acetyl-CoA with butyrate, butyryl dehydrogenase (EC 1.3.8.1), and 3-hydroxybutyryl-CoA dehydratase (EC:4.2.1.55) are down regulated in the HF comparison but not the LF comparison. While these expression differences suggest that Plin2 mice may produce more butyrate under a low-fat/high-carbohydrate diet, the relative impact for Plin2 and WT mice fed a HF diet are less clear. Further insights may be gained through the application of methods such as constraints-based modeling to examine changes in pathway flux as a consequence of enzyme expression [55].

## Discussion

Previous studies in humans and animal models have documented that diet plays a major role in driving the taxonomic structure of the intestinal microbiota and that the composition of the microbiota can have a significant impact on host health. For example, through the production of short-chain fatty acids, previous studies have shown that species of *Bacteroides*, *Alistipes*, and *Parabacteroides* can influence host body mass [56]. However, although different taxa within a microbiome can impart unique functionality, metagenomic studies have also revealed the capacity of diverse microbiomes to encode similar functional potential [57]. In this study, we applied metatranscriptomic sequence analysis to examine the potential of host genotype and diet to influence microbial community structure and function. Consistent with previous studies, we found that changes in dietary fat and/or carbohydrates had a dramatic impact on community structure. Crucially, however, we also demonstrated that deletion of a mouse lipid storage gene, *Plin2*, can significantly alter microbial gene expression, despite having minimal impact on community structure (at least in the short-term). Furthermore, although the intestinal microbiota of WT mice exhibited relatively robust and rapid transcriptional responses to changes in dietary fat/carbohydrates, the microbiota of Plin2 mice displayed even more dramatic responses, as indicated by the greater number and increased magnitude of differentially expressed pathways in this group (Fig. 3). Collectively, our results provide evidence of host gene function and diet interacting to regulate gut microbial metabolism and suggest a specific role of Plin2 host functions in integrating metabolic responses of specific bacterial taxa to HF diets.

*Plin2* is a member of the perilipin family that organizes the formation of cytoplasmic lipid droplets and contributes to the coordination of cellular and tissue lipid storage and metabolism [20]. In previous studies, we demonstrated that deletion of *Plin2* abrogates long-term deleterious effects of a high-fat diet, at least in part through limiting intestinal lipid uptake [32]. We now report that *Plin2* deletion results in altered patterns of expression of microbial enzymes responsible for directing metabolic flux through key pathways that drive the production of energy and components required for cell growth. A global consequence of these responses appears to be a general increase in pathways linked with the production of metabolic intermediates contributing to fatty acid and amino acid synthesis, in addition to other metabolites such as uracil. Importantly, data demonstrating bacterial taxon-dependent effects of *Plin2* on bacterial metabolic pathways suggest that *Plin2*-dependent modulation of host lipid metabolism is a determinant of the syntrophic metabolic optimization of gut bacteria in response to dietary substrate alteration.

The precise mechanism by which *Plin2*, or other host genes, influence gut microbial metabolism remains to be determined and may be complex. Increased quantities of fecal trigylcerides (TG) are observed in both HF-fed (relative to LF- or chow-fed) and Plin2-null (relative to WT) animals [32]. It is possible that increased availability of lipids in the intestinal lumen provides the microbial community with an excess source of energy in relation to other essential metabolites. By altering enzyme expression, the community may respond by directing metabolic flux away from the production of energy and instead into the anabolic production of biomass constituents, such as fatty acids, amino acids, and nucleotides, which are growth-limiting under conditions of lipid/energy excess. For example, through reductive carboxylation, the TCA cycle may operate in reverse, consuming energy in the form of ATP, GTP, and NADH to produce intermediates for the production of metabolically important fatty acids, such as butyrate, and amino acids, such as glutamate. Additionally, the luminal lipid concentration can influence gut osmolarity [58] and secretion of bile acids [59], both of which regulate environmental signaling systems, such as OmpR, which is involved in nutrient uptake and metabolism, as well as motility and invasion [44, 60]. Our findings that bile acid-sensitive flagellin genes [60] are differentially expressed in some bacteria of HF-fed Plin2 mice may reflect the effects of *Plin2* on bile acid production. Similarly, decreased expression of the osmotically sensitive OmpR system in HF-fed Plin2 mice is consistent with suppression of this system under low osmotic conditions [58].

The differences in expression profiles of HF-fed Plin2 mice compared with HF-fed WT mice could have arisen simply through elevated TG reaching the colons of Plin2 animals. However, this conclusion is difficult to reconcile with our finding that the genes and pathways modulated by HF vs. LF diet differed between Plin2 and WT groups (Fig. 3, noting the contrast of Diet-Plin2 with Diet-WT), as well as between HF-fed Plin2 and WT groups (Fig. 3, Geno-HF comparison). In other words, relative to WT animals, Plin2 animals did not merely exhibit an exacerbated response of similar metabolic pathways to HF feeding, distinct patterns of up- and downregulated pathways were observed between the two groups. Thus, we hypothesize that *Plin2* deletion alters the intestinal environment, and the microbiota’s transcriptional response to this environment, beyond increasing fecal TG levels; we are currently exploring other *Plin2*-dependent aspects of lipid metabolism and hepato-intestinal cross-talk, such as bile acid pool size and composition that might affect the gut microbiome in this system.

Interestingly, over the timescale of this experiment (4 day exposure to LF or HF), changes in pathway expression apparently did not selectively benefit the growth of any single taxon because no significant difference in intestinal community composition was observed between the WT or Plin2 mice. In longer experiments involving weeks of dietary exposure, we have noted compositional change in 16S rRNA datasets between WT and Plin2 mice (Frank and McManaman, unpublished). Thus, this study indicates that applying methodologies that more directly interrogate microbiome function, such as metatranscriptomic profiling, can provide a more sensitive means of detecting early or subtle changes in microbiome activity than can marker gene inventories. We note that tools such as PICRUSt [33] that seek to infer microbiota function using 16S rRNA gene abundance data may fail to recognize the potential of communities with similar taxonomic structures to exhibit significant shifts in gene expression and hence functional capacity.

Key outstanding questions include the mechanism by which changes in lipid uptake and/or metabolism by the host influence microbial gene expression patterns. By using metabolomics and stable isotope labeling, we expect that future studies will reveal how changes in enzyme expression affect the flux of metabolites through diverse metabolic pathways and thereby impact microbial growth and community function. Of particular interest would be experiments that allow the delineation of metabolites produced and consumed by the microbiome from those associated with the host. Furthermore, because the two dietary regimes examined in this study differed in both fat and carbohydrate content, additional work is needed to delineate the relative contributions of these two energy sources to the phenotypes reported in this study. Finally, longitudinal follow-up studies are needed to determine the temporal relationships between diet-induced changes in microbiome function and development of metabolic disease. Conducting these studies across developmental stages (i.e., infant, juvenile, adult, and aged mice) would also be of interest to better understand inter-generational transmission of obesity and metabolic disease risk.

## Conclusions

Obesity and its co-morbidities, such as fatty liver disease, are major global health concerns. The composition and function of the intestinal microbiome is recognized as a critical determinant of metabolic disease risk. Here, we show that under a high-fat/low-carbohydrate dietary regime, despite sharing similar microbiome community structures, the knockout of the *Plin2* gene in mice can give rise to dramatic differences in microbial gene expression profiles. Many of these changes were associated with the coordinated expression of suites of enzymes mediating consecutive reactions within a pathway, directing metabolic flux towards the production of important “biomass” constituents (i.e., amino acids, nucleotides, and other molecules required for growth). To our knowledge, this is the first demonstration of how host genotype can impact microbiome function without altering community composition, emphasizing the need to prioritize metatranscriptomic studies over 16S rRNA surveys to gain more meaningful insights into microbiome function.

## Methods

### Animal procedures

Eight-week-old male C57BL/6 (WT) and Plin2-null (Plin2) mice on the C57BL/6 background were used for all studies. The generation and characterization of Plin2-null mice have been described in detail previously [61]. The WT mice used in this study were obtained from a breeding colony maintained at the University of Colorado School of Medicine’s Center for Comparative Medicine and housed in the same room as Plin2-null mice. All mice were fed standard mice chow (2020X, Harlan Laboratories, 16% fat calories, 24% protein calories and 60% carbohydrate calories) ad libitum from weaning to 8 weeks of age, at which time they were housed individually in a metabolic monitoring system at 30 °C, the thermoneutral temperature of mice [62], for measurements of energy balance (intake and expenditure), the respiratory exchange ratio (RER), and activity levels (Columbus 8 M Oxymax) [63]. Following a 3-day adjustment period, the mice were fed nutritionally balanced high-fat (HFD; 60% fat calories, 20% protein calories, 20% carbohydrate calories, D12492) or low-fat (LFD; 10% fat calories, 20% protein calories, 70% carbohydrate calories, D12450B) diets from Research Diets Inc. (New Brunswick, NJ) ad libitum for 4 days.

### Metatranscriptomic analysis

The RiboPure-bacteria kit (Ambion, Austin, TX, USA) was used to isolate total RNA from colon contents according to the manufacturer’s protocol. Briefly, samples were collected and suspended in RNAwiz (provided in a kit) and bead beaten with zirconia beads using the Roche MagnaLyser (Roche Applied Science, Indianapolis, Indiana). Nucleic acids were recovered from the lysate by adding chloroform, centrifugation, and removal of the aqueous layer. Following ethanol precipitation, the sample were bound to a spin filter, washed, and then eluted with 50 μl of the provided elution solution. The eluted RNA was then treated with DNAse I (provided in the kit) for removal of any contaminating DNA from RNA. The final product RNA was stored in −80 °C and shipped on dry ice to the Donnelly Sequencing Centre of the University of Toronto, Canada. Ribosomal RNA depletion was performed using the Ribo-Zero™ Epidemiology kit (Epicentre Inc, Madison, WI, USA). Sequencing was performed on an Illumina NextSeq500 platform using a single high-output cartridge to generate ~ 419 million 1 × 150 bp reads. With ~ 20–30 million reads generated per sample (Table 1), rarefaction analysis revealed sufficient depth of coverage to identify the vast majority of species and enzymes present in the samples (Additional file 14: Figure S8). Sequence data is available at the NCBI Sequence Read Archive (https://www.ncbi.nlm.nih.gov/sra/) with the BioProject identifier: PRJNA379425.

Sequence reads were processed as described previously [34, 35]. In brief, we processed sequence reads by removing adaptor-contaminated and low-quality reads using Cross_Match v0.990319 (www.phrap.org) and USEARCH v7.0.1001 [64], respectively. Next, rRNA and tRNA reads were filtered using Infernal v1.1.1 [65] and host reads identified through BWA v0.7.5a [66] and BLAT v35 [67] sequence similarity searches against a database of mouse transcripts (ENSEMBL release 78 [68]). Putative mRNA reads were then assembled using the Trinity v2.1.1 de novo assembler [69], and resultant contigs, as well as unassembled reads, were assigned to microbial transcripts using a tiered set of sequence similarity searches against a database of sequenced microbial genomes (downloaded from NCBI June 2015) using BWA and BLAT, as well as the protein non-redundant database (downloaded from NCBI July 2015) using DIAMOND v0.7.5a [38]. The expression level of a microbial transcript is then based on the number of sequence reads mapped to that transcript. Consistent with previous studies [70], we first filtered for transcripts expressed at low levels, defined here as those with < 5 mapped reads (representing ~ 6.5% of all mapped reads), and then normalized expression levels of transcripts as reads per kilobase per million mapped reads (RPKM; [71]). Where no expression was observed, RPKM values were set to 0.

### Non-parametric permutational multivariate analysis

For each dietary/genotype comparison, in addition to examining differences in the overall distribution of microbiome composition, we also investigated changes in the abundance of each taxon, defined as the sum of RPKM values of transcripts assigned to that taxon. For the former, the influence of only a single factor was investigated in the analysis, i.e., genotype (Plin2 vs. WT) or diet (HF vs. LF). For each taxon, we estimated the influence of two independent factors, genotype and diet, on one dependent variable, i.e., relative expression as defined by RPKM values. Comparisons were performed using the non-parametric permutational multivariate analysis of variance test (PERMANOVA; [72]) to assess the difference in microbiome composition between different genotypes or diets. PERMANOVA was implemented through the *f_npManov* function of MATLAB (R2015a, The MathWorks Inc., Natick, MA, USA) toolbox *Fathom* [73], using 100,000 replicate label permutations and adjusting *p* values with the Benjamini-Hochberg procedure [74]. The cutoff of the adjusted *p* value was set as 0.05.

### Principle component analysis

To reveal the correlation of the overall expression distributions relating to taxa, transcripts, differentially expressed transcripts, enzymes, and pathways across the 16 samples, we applied principal component analysis (PCA) using the *pca* function from MATLAB (R2015a, The MathWorks Inc., Natick, MA, USA). In addition, we used PERMANOVA to test how well the first and the second principle components separate sample types (e.g., Plin2-HF vs. WT-HF).

### Biodiversity analysis

We used four biodiversity indices, i.e., Shannon entropy index (Shannon), Fisher’s alpha index (F-alpha), Chao1 index (Chao1), and Simpson index (Simpson) to examine biodiversity distributions of our data. Chao1 values were calculated using EstimateS v 9.1.0 [75] with 100 bootstrap replicates. Other indices were calculated using the vegan package v2.4.3 [76] in R v3.4.0 [77].

### Gene set enrichment analysis

To test if taxonomic categories and KEGG-defined pathways were enriched with either significantly differentially expressed transcripts or enzymes, gene set enrichment analyses were performed using a hypergeometric test with a minimum of two genes per gene set. In these analyses, to ensure consistency across sample comparison, we examined enrichment relative to the total pool of all transcripts identified across all 16 samples. We used a false discovery rate (FDR) adjustment with the Benjamini-Hochberg procedure to correct *p* values. Hypergeometric tests were performed using the *hygecdf* and *mafdr* functions from MATLAB (R2015a, The MathWorks Inc., Natick, MA, USA) with a FDR cutoff of 0.05.

### Expression fold change

Given a transcript or enzyme *g*, its expression fold change (*eFC*), for a pairwise comparison between sample 1 and 2, was calculated as:

$$ {\displaystyle \begin{array}{l}{eFC}_{12}\left\{\begin{array}{c}\hfill \operatorname{sign}{\left({r}_2-{r}_1\right)}^{\ast}\frac{\max \left({r}_1,{r}_2\right)}{\min \left({r}_1,{r}_2\right)},\min \left({r}_1,{r}_2\right)>0\hfill \\ {}\hfill \kern2.1em {r}_2-{r}_1,\min \left({r}_1,{r}_2\right)=0\hfill \end{array}\right.\\ {}\end{array}} $$

where *r*_1_ is the RPKM value of a transcript or enzyme in sample 1 (note several transcripts may be assigned the same enzyme (EC number)), while *r*_2_ is the RPKM of the transcript or enzyme in sample 2. *eFC*_12_ > 0 indicates the transcript or enzyme *g* is upregulated in sample 2, while *eFC*_12_ < 0 indicates *g* is downregulated in sample 2. The average *eFC* (*aeFC*) of an enzyme, for a sample-wise comparison between sample 1 and 2, is calculated as:

$$ {aeFC}_{12}={eFC}_{12}/n $$

Where *n* is the total number of transcripts mapped to this enzyme in that sample. Pathway visualizations of enzyme eFCs were performed using Cytoscape v3.4.0 [78] with pathways downloaded in KGML format from KEGG [79].

### Differential expression analysis of transcripts and enzymes

Differential expression analysis of mapped transcripts for different sample-wise comparisons was performed using DESeq [39]. Since our interest is focused on comparisons between different genotypes or diets, only four combinations were explored, i.e., Plin2-HF vs. WT-HF (Geno-HF), Plin2-LF vs. WT-LF (Geno-LF), Plin2-HF vs. Plin2-LF (Diet-Plin2), and WT-HF vs. WT-LF (Diet-WT). For each pairwise comparison, we defined significantly differentially expressed transcripts (sig. transcripts) as those with *q* values < 0.05 (using the Benjamini-Hochberg procedure to correct *p* values) and Log_2_ (*eFC*) > 2. Across all comparisons, 3777 unique sig. transcripts were identified. Enzymes are defined as significantly differentially expressed if at least one sig. transcript is mapped to that enzyme.

## Additional files

Additional file 1: Table S1. Summary statistics of sequencing. (XLSX 19 kb)

Additional file 2: Table S2. Expression values (RPKM) for each of the 57,736 transcripts identified across all samples. (XLSX 35991 kb)

Additional file 3: Figure S1. Principal component analysis for four data types (Taxa, Enzymes, Significant Differentially Expressed Transcripts and Metabolic Pathways). With each plot, *p* values (< 0.05) are provided indicating significant differences in clustering between each of the four pairwise comparisons. (PDF 912 kb)

Additional file 4: Table S3. Expression values (RPKM) of 1344 transcripts displaying differential expression across at least one of the four pairwise comparisons (Plin2-HF vs. WT-HF, Plin2-HF vs. Plin2-LF, Plin2-LF vs. WT-LF, and WT-HF vs. WT-LF). (XLSX 799 kb)

Additional file 5: Table S4. Breakdown of genomes, transcripts, and average RPKM per taxon across all samples. (XLSX 17 kb)

Additional file 6: Table S5. Enrichment of sig. enzymes in 144 KEGG pathways for four pairwise comparisons (Plin2-HF vs. WT-HF, Plin2-HF vs. Plin2-LF, Plin2-LF vs. WT-LF, and WT-HF vs. WT-LF). (XLSX 41 kb)

Additional file 7: Figure S2. Comparison of TCA cycle enzyme expression in between sample types. Three comparisons are shown: (A) Plin2-LF vs. WT-LF; (B) Plin2-HF vs. Plin2-LF; (C) WT-HF vs. WT-LF. Circular nodes indicate enzymes, with size indicating relative difference in expression between sample types and color indicating direction of change (see inset key). Associated heatmaps indicate global changes in expression for each enzyme, in addition to taxon-specific changes in expression for each of the 17 defined taxa colored according to phylum. (PDF 1246 kb)

Additional file 8: Figure S3. Comparison of glycolysis pathway enzyme expression between sample types. Three comparisons are shown: (A) Plin2-LF vs. WT-LF; (B) Plin2-HF vs. Plin2-LF; (C) WT-HF vs. WT-LF. Circular nodes indicate enzymes, with size indicating relative difference in expression between sample types and color indicating direction of change (see inset key). Associated heatmaps indicate global changes in expression for each enzyme, in addition to taxon-specific changes in expression for each of the 17 defined taxa colored according to phylum. (PDF 1138 kb)

Additional file 9: Figure S4 Genotype-based comparisons of pantothenate pathway enzyme expression. Two comparisons are shown: (A) Plin2-HF vs. WT-HF and (B) Plin2-LF vs. WT-LF. Circular nodes indicate enzymes, with size indicating relative difference in expression between sample types and color indicating direction of change (see inset key). Associated heatmaps indicate global changes in expression for each enzyme, in addition to taxon-specific changes in expression for each of the 17 defined taxa colored according to phylum. The following abbreviations are used: 5,6-dh-uracil (5,6-dihydro-uracil), *N*-cm-β-alanine (*N*-carbamoyl-β-alanine), *N*-pt-Cys (*N*-pantothenoyl-cysteine), and (R)-4′-P-pt-L-Cys ((R)-4′-phospho-pantothenoyl-l-cysteine. (PDF 1370 kb)

Additional file 10: Figure S5. Diet-based comparisons of enzyme expression in pantothenate pathway. Two comparisons are shown: (A) Plin2-HF vs. Plin2-LF and (B) WT-HF vs. WT-LF. Circular nodes indicate enzymes, with size indicating relative difference in expression between sample types and color indicating direction of change (see inset key). Associated heatmaps indicate global changes in expression for each enzyme, in addition to taxon-specific changes in expression for each of the 17 defined taxa colored according to phylum. The following abbreviations are used: 5,6-dh-uracil (5,6-dihydro-uracil), *N*-cm-β-alanine (*N*-carbamoyl-β-alanine), *N*-pt-Cys (*N*-pantothenoyl-cysteine), and (R)-4′-P-pt-L-Cys ((R)-4′-phospho-pantothenoyl-l-cysteine. (PDF 1119 kb)

Additional file 11: Figure S6. Genotype-based comparisons of enzyme expression in butanoate pathway. Two comparisons are shown: (A) Plin2-HF vs. WT-HF and (B) Plin2-LF vs. WT-LF. Circular nodes indicate enzymes, with size indicating relative difference in expression between sample types and color indicating direction of change (see inset key). Associated heatmaps indicate global changes in expression for each enzyme, in addition to taxon-specific changes in expression for each of the 17 defined taxa colored according to phylum. The following abbreviations are used: 3B–CoA (3-butenoyl-CoA), 4HB-CoA (4-hydroxy-butanoyl-CoA), C-CoA (crotonoyl-CoA), G-CoA (glutaconyl-CoA), 3HB-CoA (3-hydroxybutanoyl-CoA), and HMG-CoA (hydroxy-3-methylglutaryl-CoA). (PDF 1234 kb)

Additional file 12: Figure S7. Diet-based comparisons of enzyme expression in butanoate pathway. Two comparisons are shown: (A) Plin2-HF vs. Plin2-LF and (B) WT-HF vs. WT-LF. Circular nodes indicate enzymes, with size indicating relative difference in expression between sample types and color indicating direction of change (see inset key). Associated heatmaps indicate global changes in expression for each enzyme, in addition to taxon-specific changes in expression for each of the 17 defined taxa colored according to phylum. The following abbreviations are used: 3B–CoA (3-butenoyl-CoA), 4HB-CoA (4-hydroxy-butanoyl-CoA), C-CoA (crotonoyl-CoA), G-CoA (glutaconyl-CoA), 3HB-CoA (3-hydroxybutanoyl-CoA), HMG-CoA (hydroxy-3-methylglutaryl-CoA). (PDF 1262 kb)

Additional file 13: Table S6. Expression fold change (eFC) values of sig. enzymes for four pairwise comparisons (Plin2-HF vs. WT-HF, Plin2-HF vs. Plin2-LF, Plin2-LF vs. WT-LF and WT-HF vs. WT-LF) (XLSX 80 kb)

Additional file 14: Figure S8. Rarefaction analysis of annotated mRNA reads. Recovery of species (A) and enzymes (B) with increasing numbers of annotated mRNA reads (reads mapped to known transcripts) indicate that sequencing depth for each sample was sufficient to recover the vast majority of species and enzymes present within each of the 16 samples. Rarefaction analysis was performed using R. (PDF 16825 kb)

## References

[1] NCD Risk Factor Collaboration (NCD-RisC) (2016). Trends in adult body-mass index in 200 countries from 1975 to 2014: a pooled analysis of 1698 population-based measurement studies with 19.2 million participants. Lancet.

[2] World Health Organization: Obesity and overweight [Fact sheet]. 2016.

[3] Ussar S, Griffin Nicholas W, Bezy O, Fujisaka S, Vienberg S, Softic S, Deng L, Bry L, Gordon Jeffrey I, Kahn CR (2015). Interactions between gut microbiota, host genetics and diet modulate the predisposition to obesity and metabolic syndrome. Cell Metab..

[4] Parekh PJ, Balart LA, Johnson DA (2015). The Influence of the gut microbiome on obesity, metabolic syndrome and gastrointestinal disease. Clin. Transl Gastroenterol.

[5] Catenacci VA, Hill JO, Wyatt HR (2009). The obesity epidemic. Clin. Chest Med..

[6] Wlodarska M, Kostic Aleksandar D, Xavier Ramnik J (2015). An Integrative View of Microbiome-Host Interactions in Inflammatory Bowel Diseases. Cell Host Microbe.

[7] Qin J, Li Y, Cai Z, Li S, Zhu J, Zhang F, Liang S, Zhang W, Guan Y, Shen D (2012). A metagenome-wide association study of gut microbiota in type 2 diabetes. Nature.

[8] Unger RH, Clark GO, Scherer PE, Orci L (1801). Lipid homeostasis, lipotoxicity and the metabolic syndrome. Biochimica et biophysica acta.

[9] Frank DN, Zhu W, Sartor RB, Li E (2011). Investigating the biological and clinical significance of human dysbioses. Trends Microbiol.

[10] Tremaroli V, Backhed F (2012). Functional interactions between the gut microbiota and host metabolism. Nature.

[11] Donohoe DR, Garge N, Zhang X, Sun W, O'Connell TM, Bunger MK, Bultman SJ (2011). The microbiome and butyrate regulate energy metabolism and autophagy in the mammalian colon. Cell Metab.

[12] Scheppach W (1994). Effects of short chain fatty acids on gut morphology and function. Gut.

[13] David LA, Maurice CF, Carmody RN, Gootenberg DB, Button JE, Wolfe BE, Ling AV, Devlin AS, Varma Y, Fischbach MA (2014). Diet rapidly and reproducibly alters the human gut microbiome. Nature.

[14] Sonnenburg JL, Backhed F (2016). Diet-microbiota interactions as moderators of human metabolism. Nature.

[15] Beilstein F, Carrière V, Leturque A, Demignot S (2016). Characteristics and functions of lipid droplets and associated proteins in enterocytes. Exp Cell Res..

[16] Wilfling F, Haas JT, Walther TC, Farese RV (2014). Lipid droplet biogenesis. Curr Opin Cell Biol.

[17] Khor VK, Shen WJ, Kraemer FB (2013). Lipid droplet metabolism. Curr. Opin. Clin. Nutr. Metab. Care.

[18] Walther TC, Farese RV (2012). Lipid droplets and cellular lipid metabolism. Annu Rev Biochem.

[19] Brasaemle DL, Wolins NE (2012). Packaging of fat: an evolving model of lipid droplet assembly and expansion. J Biol Chem.

[20] Greenberg AS, Coleman RA, Kraemer FB, McManaman JL, Obin MS, Puri V, Yan QW, Miyoshi H, Mashek DG (2011). The role of lipid droplets in metabolic disease in rodents and humans. J Clin Invest.

[21] Libby AE, Bales E, Orlicky DJ, McManaman JL (2016). Perilipin-2 deletion impairs hepatic lipid accumulation by interfering with sterol regulatory element-binding protein (SREBP) activation and altering the hepatic lipidome. J Biol Chem.

[22] Greenberg AS, Coleman RA, Kraemer FB, McManaman JL, Obin MS, Puri V, Yan Q-W, Miyoshi H, Mashek DG (2011). The role of lipid droplets in metabolic disease in rodents and humans. J. Clin. Invest..

[23] Kimmel AR, Sztalryd C (2016). The perilipins: major cytosolic lipid droplet-associated proteins and their roles in cellular lipid storage, mobilization, and systemic homeostasis. Annu Rev Nutr.

[24] Smith CE, Ordovas JM (2012). Update on perilipin polymorphisms and obesity. Nutr Rev.

[25] Tai ES, Ordovas JM (2007). The role of perilipin in human obesity and insulin resistance. Curr. Opin. Lipidol..

[26] Brasaemle DL (2007). Thematic review series: adipocyte biology. The perilipin family of structural lipid droplet proteins: stabilization of lipid droplets and control of lipolysis. J. Lipid Res.

[27] McManaman JL, Bales ES, Orlicky DJ, Jackman M, MacLean PS, Cain S, Crunk AE, Mansur A, Graham CE, Bowman TA (2013). Perilipin-2-null mice are protected against diet-induced obesity, adipose inflammation, and fatty liver disease. J. Lipid Res.

[28] Orlicky DJ, Monks J, Stefanski AL, McManaman JL (2013). Dynamics and molecular determinants of cytoplasmic lipid droplet clustering and dispersion. PLoS One.

[29] Varela LM, Lopez S, Ortega-Gomez A, Bermudez B, Buers I, Robenek H, Muriana FJ, Abia R: Postprandial triglyceride-rich lipoproteins regulate perilipin-2 and perilipin-3 lipid-droplet-associated proteins in macrophages. J. Nutr. Biochem. 2015, 26(4):327-336. 10.1016/j.jnutbio.2014.11.007 25595097

[30] Carr RM, Peralta G, Yin X, Ahima RS (2014). Absence of perilipin 2 prevents hepatic steatosis, glucose intolerance and ceramide accumulation in alcohol-fed mice. PLoS One.

[31] Kaushik S, Cuervo AM (2015). Degradation of lipid droplet-associated proteins by chaperone-mediated autophagy facilitates lipolysis. Nat Cell Biol.

[32] Frank DN, Bales ES, Monks J, Jackman MJ, MacLean PS, Ir D, Robertson CE, Orlicky DJ, McManaman JL (2015). Perilipin-2 modulates lipid absorption and microbiome responses in the mouse intestine. PLoS One.

[33] Langille MGI, Zaneveld J, Caporaso JG, McDonald D, Knights D, Reyes JA, Clemente JC, Burkepile DE, Vega Thurber RL, Knight R (2013). Predictive functional profiling of microbial communities using 16S rRNA marker gene sequences. Nat Biotech.

[34] Xiong X, Frank DN, Robertson CE, Hung SS, Markle J, Canty AJ, McCoy KD, Macpherson AJ, Poussier P, Danska JS *et al*: Generation and analysis of a mouse intestinal metatranscriptome through Illumina based RNA-sequencing. PloS one 2012, 7(4):e36009. 10.1371/journal.pone.0036009 PMC3338770 22558305

[35] Jiang Y, Xiong X, Danska J, Parkinson J (2016). Metatranscriptomic analysis of diverse microbial communities reveals core metabolic pathways and microbiome-specific functionality. Microbiome.

[36] Li H, Durbin R (2010). Fast and accurate long-read alignment with Burrows-Wheeler transform. Bioinformatics.

[37] Kent WJ (2002). BLAT—the BLAST-like alignment tool. Genome Res.

[38] Buchfink B, Xie C, Huson DH (2015). Fast and sensitive protein alignment using DIAMOND. Nat Methods.

[39] Anders S, Huber W (2010). Differential expression analysis for sequence count data. Genome Biol.

[40] Galperin MY (2006). Structural classification of bacterial response regulators: diversity of output domains and domain combinations. J Bacteriol.

[41] Cai SJ, Inouye M (2002). EnvZ-OmpR interaction and osmoregulation in Escherichia coli. J Biol Chem.

[42] Shin S, Park C (1995). Modulation of flagellar expression in Escherichia coli by acetyl phosphate and the osmoregulator OmpR. J Bacteriol.

[43] Shimizu K (2014). Regulation systems of bacteria such as Escherichia coli in response to nutrient limitation and environmental stresses. Metabolites.

[44] Cho BK, Knight EM, Palsson BO (2006). Transcriptional regulation of the fad regulon genes of Escherichia coli by ArcA. Microbiology.

[45] Hu Y, Wang Y, Ding L, Lu P, Atkinson S, Chen S (2009). Positive regulation of flhDC expression by OmpR in Yersinia pseudotuberculosis. Microbiology.

[46] Lodes MJ, Cong Y, Elson CO, Mohamath R, Landers CJ, Targan SR, Fort M, Hershberg RM (2004). Bacterial flagellin is a dominant antigen in Crohn disease. J. Clin. Invest..

[47] Vijay-Kumar M, Gewirtz AT (2009). Role of flagellin in Crohn's disease: emblematic of the progress and enigmas in understanding inflammatory bowel disease. Inflamm. Bowel Dis.

[48] Gewirtz AT, Navas TA, Lyons S, Godowski PJ, Madara JL (2001). Cutting edge: bacterial flagellin activates basolaterally expressed TLR5 to induce epithelial proinflammatory gene expression. J. Immunol. (Baltimore, Md: 1950).

[49] Vital M, Howe AC, Tiedje JM (2014). Revealing the bacterial butyrate synthesis pathways by analyzing (meta) genomic data. mBio.

[50] Schoenhofen IC, McNally DJ, Brisson J-R, Logan SM (2006). Elucidation of the CMP-pseudaminic acid pathway in Helicobacter pylori: synthesis from UDP-N-acetylglucosamine by a single enzymatic reaction. Glycobiology.

[51] Schirm M, Soo EC, Aubry AJ, Austin J, Thibault P, Logan SM (2003). Structural, genetic and functional characterization of the flagellin glycosylation process in Helicobacter pylori. Mol. Microbiol..

[52] Ishiyama N, Creuzenet C, Miller WL, Demendi M, Anderson EM, Harauz G, Lam JS, Berghuis AM (2006). Structural studies of FlaA1 from Helicobacter pylori reveal the mechanism for inverting 4,6-dehydratase activity. J. Biophys. Chem..

[53] Kanehisa M, Goto S, Hattori M, Aoki-Kinoshita KF, Itoh M, Kawashima S, Katayama T, Araki M, Hirakawa M (2006). From genomics to chemical genomics: new developments in KEGG. Nucleic Acids Res..

[54] Fischbach MA, Sonnenburg JL (2011). Eating for two: how metabolism establishes interspecies interactions in the gut. Cell Host Microbe.

[55] Khandelwal RA, Olivier BG, Roling WF, Teusink B, Bruggeman FJ (2013). Community flux balance analysis for microbial consortia at balanced growth. PLoS One.

[56] Ridaura VK, Faith JJ, Rey FE, Cheng J, Duncan AE, Kau AL, Griffin NW, Lombard V, Henrissat B, Bain JR (2013). Gut microbiota from twins discordant for obesity modulate metabolism in mice. Science.

[57] Human Microbiome Project C (2012). Structure, function and diversity of the healthy human microbiome. Nature.

[58] Overduin J, Tylee TS, Frayo RS, Cummings DE (2014). Hyperosmolarity in the small intestine contributes to postprandial ghrelin suppression. Am J Physiol Gastrointest Liver Physiol.

[59] Reddy BS (1981). Diet and excretion of bile acids. Cancer Research.

[60] Hamner S, McInnerney K, Williamson K, Franklin MJ, Ford TE (2013). Bile salts affect expression of Escherichia coli O157:H7 genes for virulence and iron acquisition, and promote growth under iron limiting conditions. PLoS One.

[61] McManaman JL, Bales ES, Orlicky DJ, Jackman M, MacLean PS, Cain S, Crunk AE, Mansur A, Graham CE, Bowman TA (2013). Perilipin-2-null mice are protected against diet-induced obesity, adipose inflammation, and fatty liver disease. Journal of lipid research.

[62] Speakman JR, Keijer J (2012). Not so hot: optimal housing temperatures for mice to mimic the thermal environment of humans. Mol Metab.

[63] Wahlig JL, Bales ES, Jackman MR, Johnson GC, McManaman JL, Maclean PS (2012). Impact of high-fat diet and obesity on energy balance and fuel utilization during the metabolic challenge of lactation. Obesity (Silver Spring, Md).

[64] Edgar RC (2010). Search and clustering orders of magnitude faster than BLAST. Bioinformatics.

[65] Nawrocki EP, Eddy SR (2013). Infernal 1.1: 100-fold faster RNA homology searches. Bioinformatics.

[66] Li H, Durbin R (2009). Fast and accurate short read alignment with Burrows-Wheeler transform. Bioinformatics.

[67] Kent WJ. BLAT: the BLAST-like alignment tool. Genome Res. 2002;12 10.1101/gr.229202 PMC187518 11932250

[68] Flicek P, Amode MR, Barrell D, Beal K, Billis K, Brent S, Carvalho-Silva D, Clapham P, Coates G, Fitzgerald S (2014). Ensembl 2014. Nucleic Acids Res.

[69] Grabherr MG, Haas BJ, Yassour M, Levin JZ, Thompson DA, Amit I, Adiconis X, Fan L, Raychowdhury R, Zeng Q (2011). Full-length transcriptome assembly from RNA-Seq data without a reference genome. Nat Biotech.

[70] Chen Y, Lun ATL, Smyth GK (2016). From reads to genes to pathways: differential expression analysis of RNA-Seq experiments using Rsubread and the edgeR quasi-likelihood pipeline. F1000Res.

[71] Mortazavi A, Williams BA, McCue K, Schaeffer L, Wold B (2008). Mapping and quantifying mammalian transcriptomes by RNA-Seq. Nat Methods.

[72] Anderson MJ (2001). A new method for non-parametric multivariate analysis of variance. Austral Ecol.

[73] Jones DL (2015). Fathom Toolbox for Matlab: software for multivariate ecological and oceanographic data analysis.

[74] Benjamini Y, Hochberg Y (1995). Controlling the false discovery rate: a practical and powerful approach to multiple testing. Journal of the Royal Statistical Society Series B (Methodological).

[75] Colwell RK (2005). EstimateS: Statistical estimation of species richness and shared species from samples.

[76] Dixon P (2003). VEGAN, a package of R functions for community ecology. Journal of Vegetation Science.

[77] R Development Core Team (2010). R: A language and environment for statistical computing.

[78] Shannon P, Markiel A, Ozier O, Baliga NS, Wang JT, Ramage D, Amin N, Schwikowski B, Ideker T (2003). Cytoscape: a software environment for integrated models of biomolecular interaction networks. Genome Res.

[79] Kanehisa M, Goto S (2000). KEGG: kyoto encyclopedia of genes and genomes. Nucleic Acids Res.

