# Supplementary figures and images for: Perilipin-2 modulates dietary fat-induced microbial global gene expression profiles in the mouse intestine
Source: Microbiome. 2017 Sep 6;5:117. doi: 10.1186/s40168-017-0327-x (PMC5588750; doi:10.1186/s40168-017-0327-x)

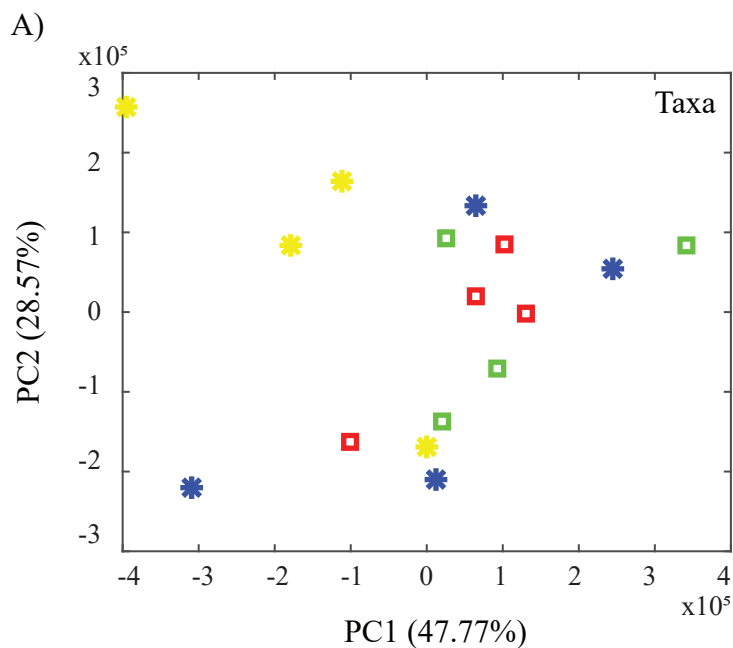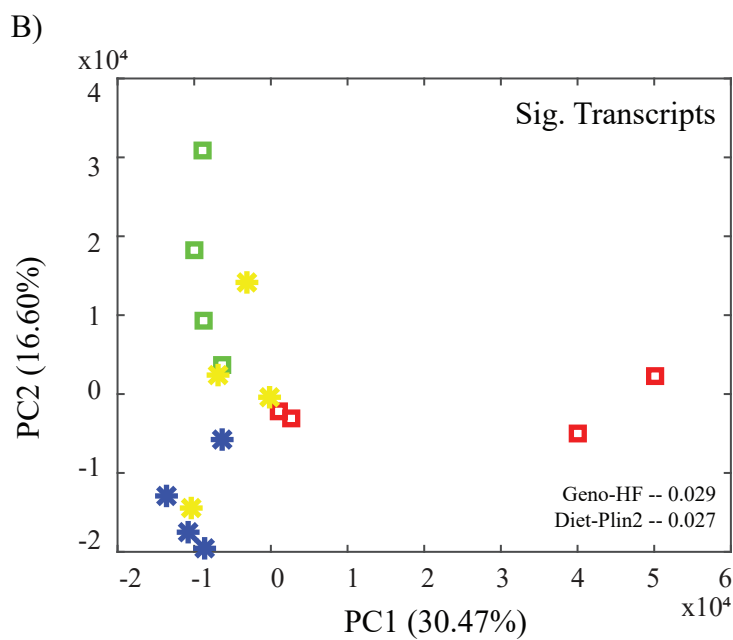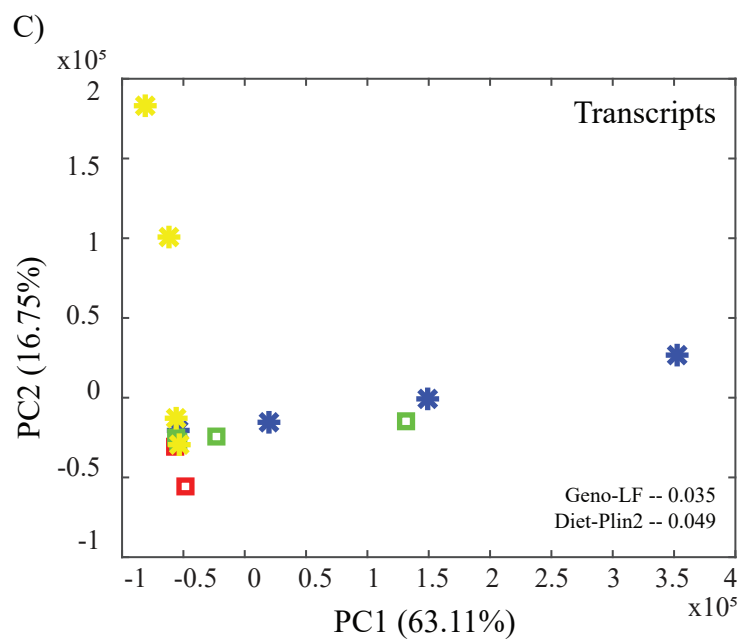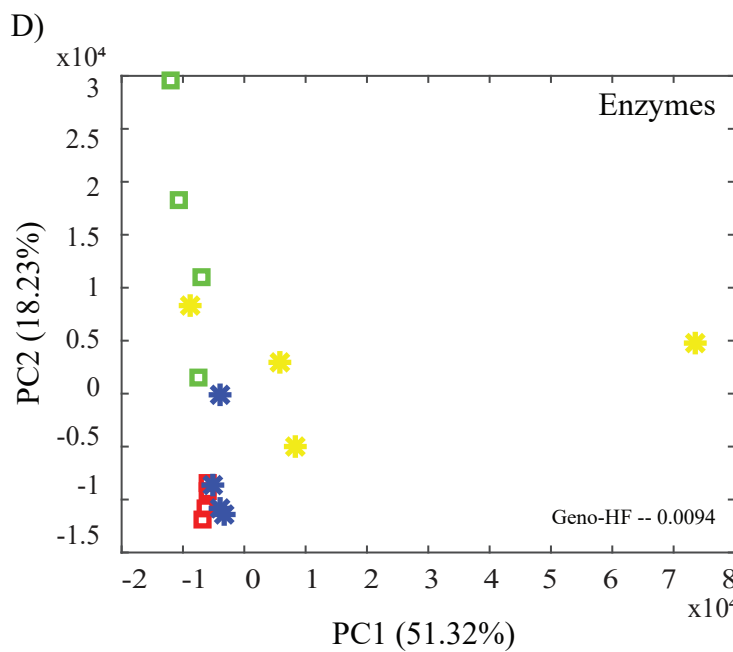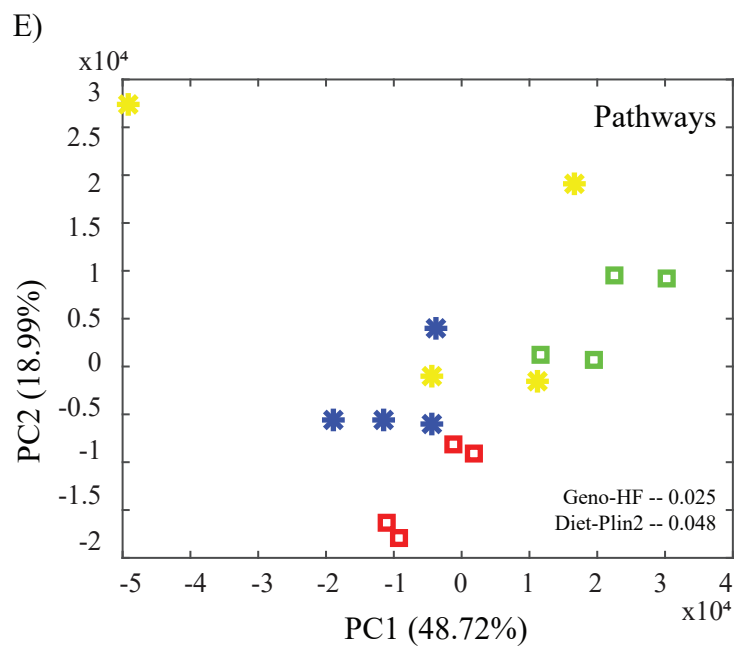

Supplement: Supplementary file 3 — Principal component analysis for four data types (Taxa, Enzymes, Significant Differentially Expressed Transcripts and Metabolic Pathways). With each plot, p values (< 0.05) are provided indicating significant differences in clustering between each of the four pairwise comparisons. (PDF 912 kb) [file 40168_2017_327_MOESM3_ESM.pdf]

# Citrate cycle (TCA cycle)

A) Geno-LF (Plin2-LF vs. WT-LF)

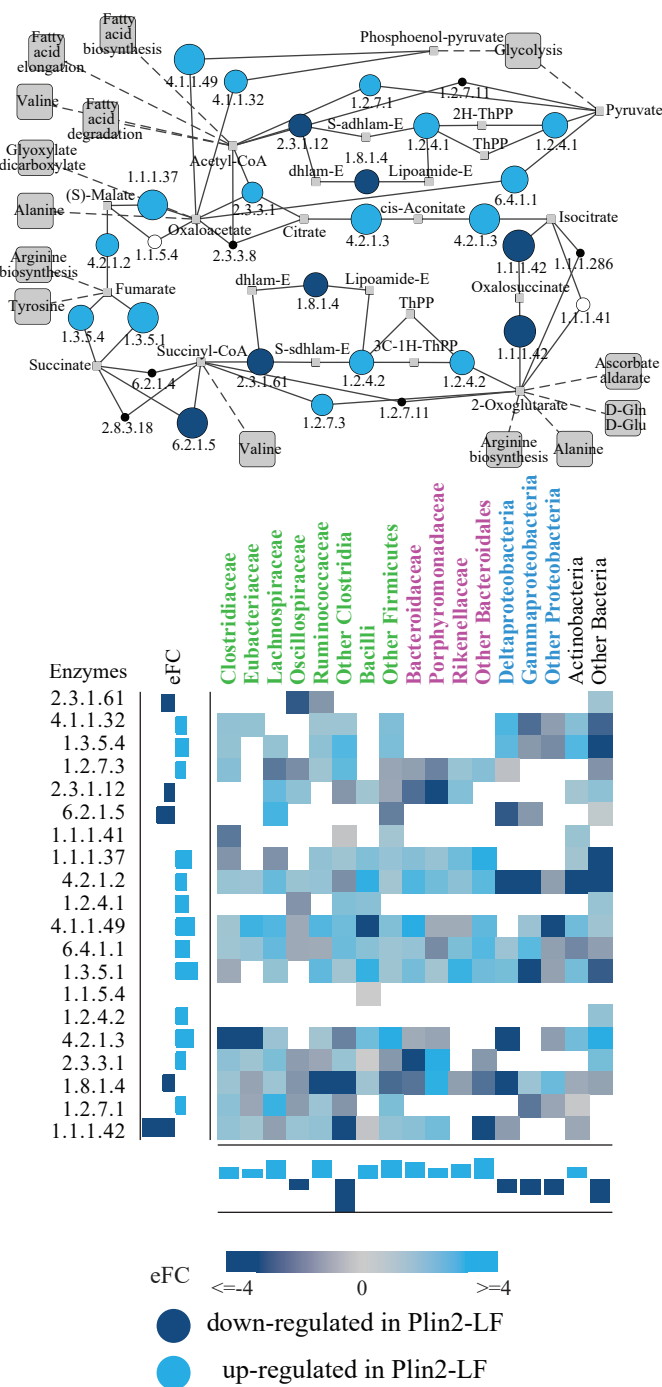

B) Diet-Plin2 (Plin2-HF vs. Plin2-LF)

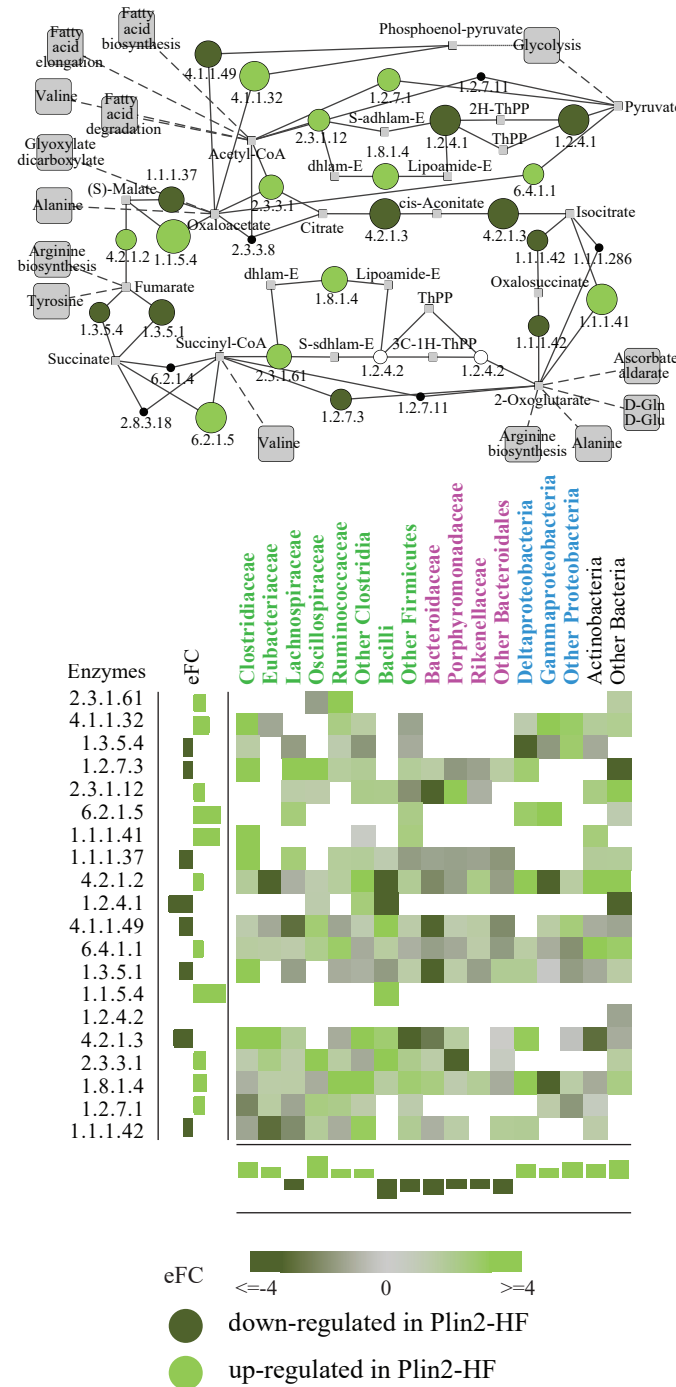

C) Diet-WT (WT-HF vs. WT-LF)

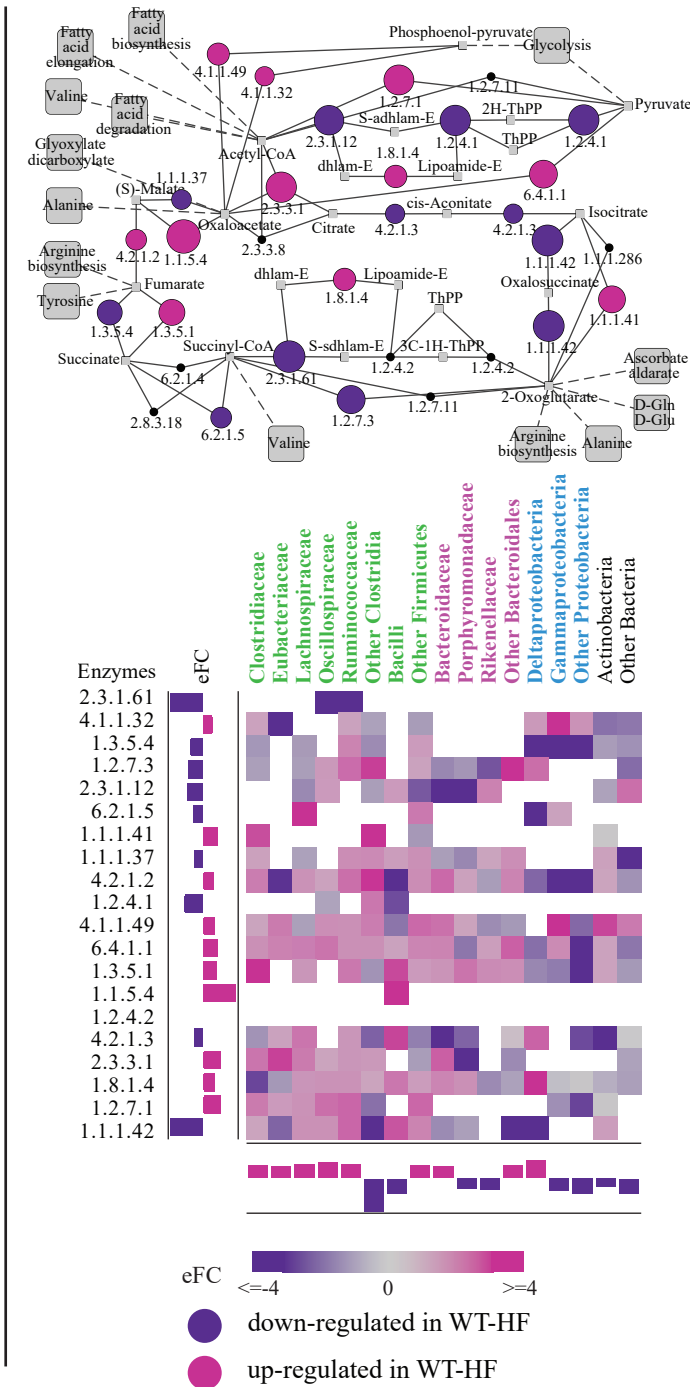

Supplement: Supplementary file 7 — Comparison of TCA cycle enzyme expression in between sample types. Three comparisons are shown: (A) Plin2-LF vs. WT-LF; (B) Plin2-HF vs. Plin2-LF; (C) WT-HF vs. WT-LF. Circular nodes indicate enzymes, with size indicating relative difference in expression between sample types and color indicating direction of change (see inset key). Associated heatmaps indicate global changes in expression for each enzyme, in addition to taxon-specific changes in expression for each of the 17 defined taxa colored according to phylum. (PDF 1246 kb) [file 40168_2017_327_MOESM7_ESM.pdf]

# Pantothenate and CoA biosynthesis

A) Geno-HF (Plin2-HF vs. WT-HF)

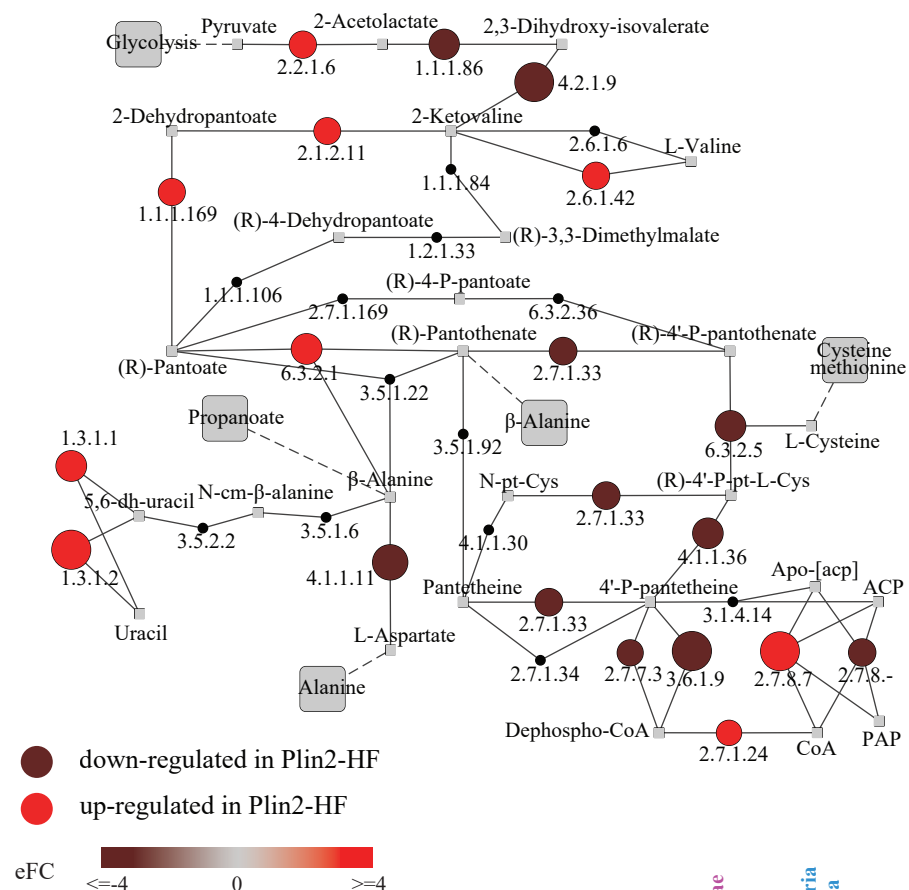

B) Geno-LF (Plin2-LF vs. WT-LF)

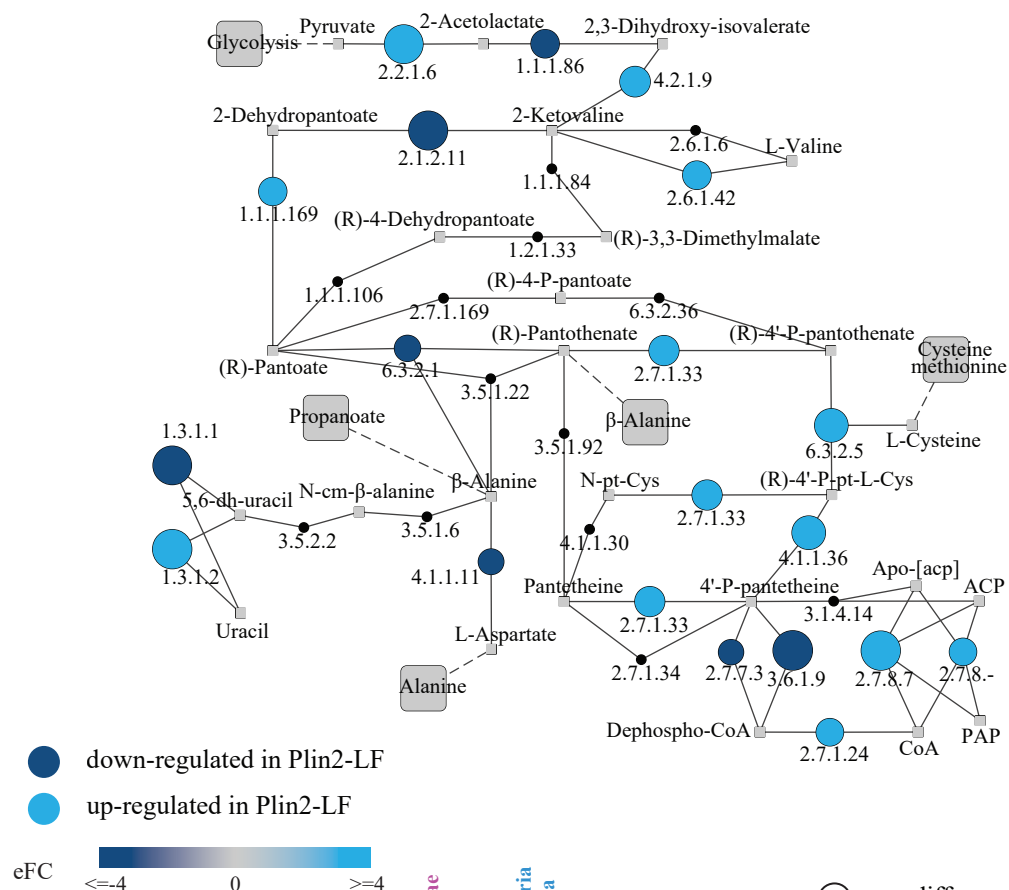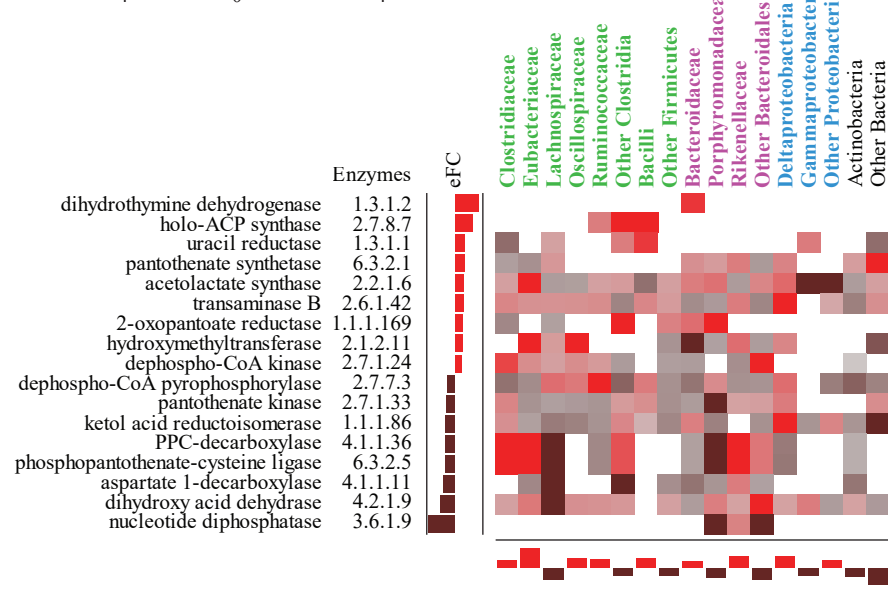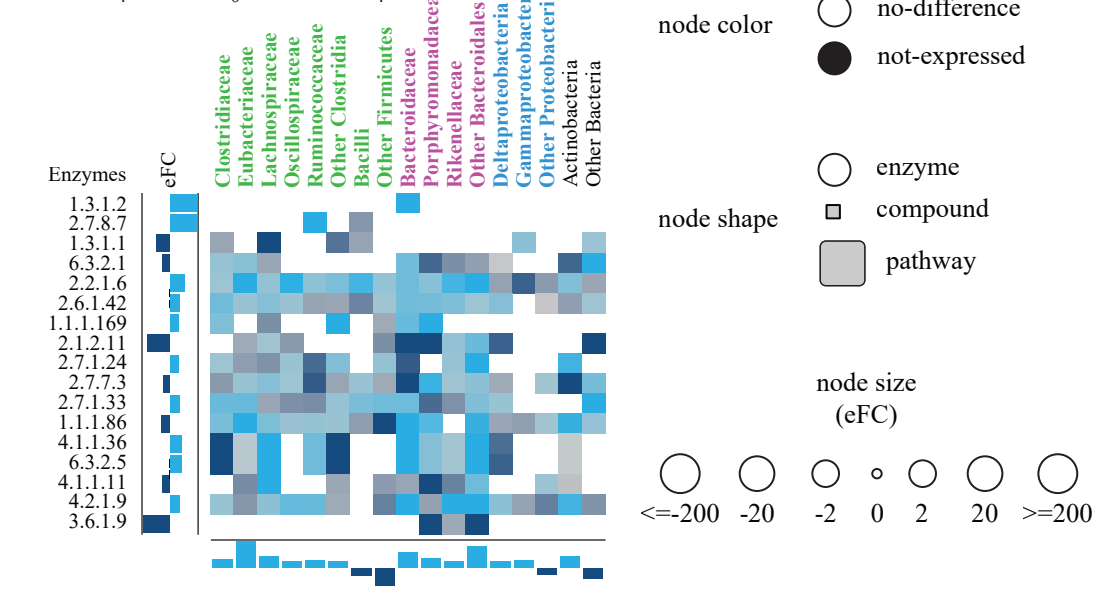

Supplement: Supplementary file 8 — Comparison of glycolysis pathway enzyme expression between sample types. Three comparisons are shown: (A) Plin2-LF vs. WT-LF; (B) Plin2-HF vs. Plin2-LF; (C) WT-HF vs. WT-LF. Circular nodes indicate enzymes, with size indicating relative difference in expression between sample types and color indicating direction of change (see inset key). Associated heatmaps indicate global changes in expression for each enzyme, in addition to taxon-specific changes in expression for each of the 17 defined taxa colored according to phylum. (PDF 1138 kb) [file 40168_2017_327_MOESM8_ESM.pdf]

# Pantothenate and CoA biosynthesis

A) Diet-Plin2 (Plin2-HF vs. Plin2-LF)

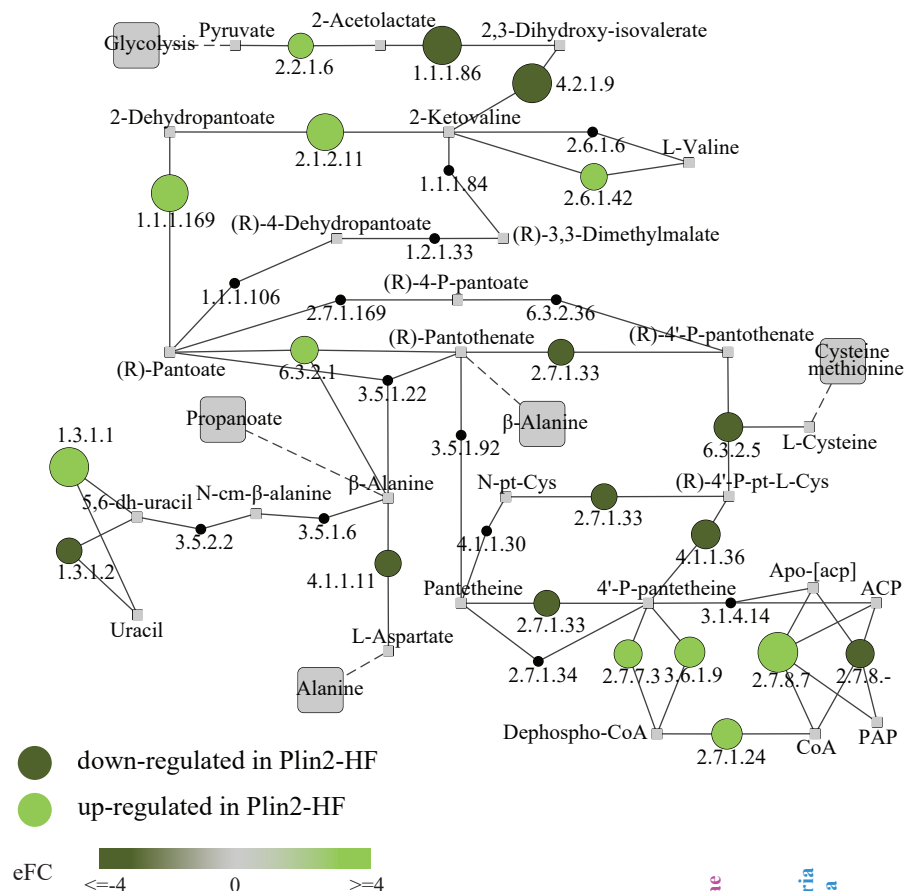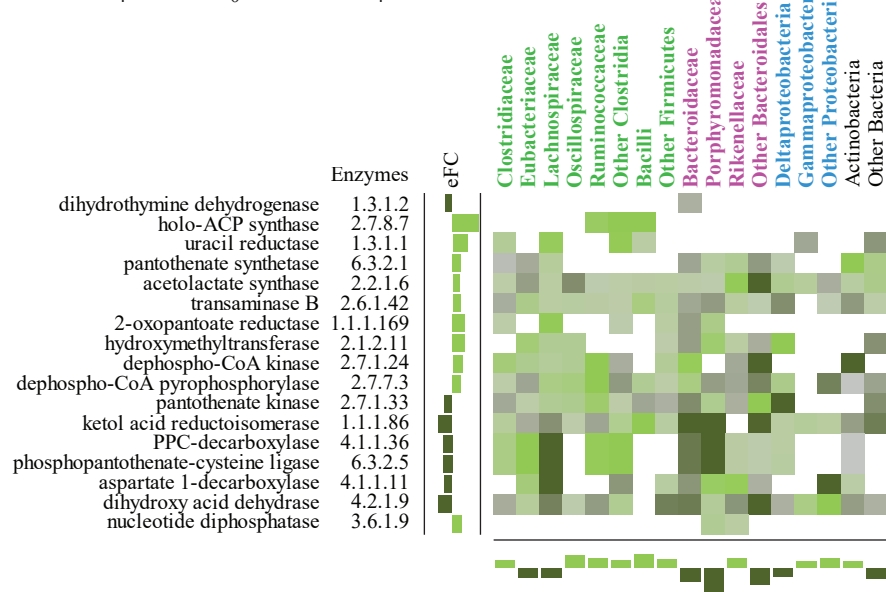

B) Diet-WT (WT-HF vs. WT-LF)

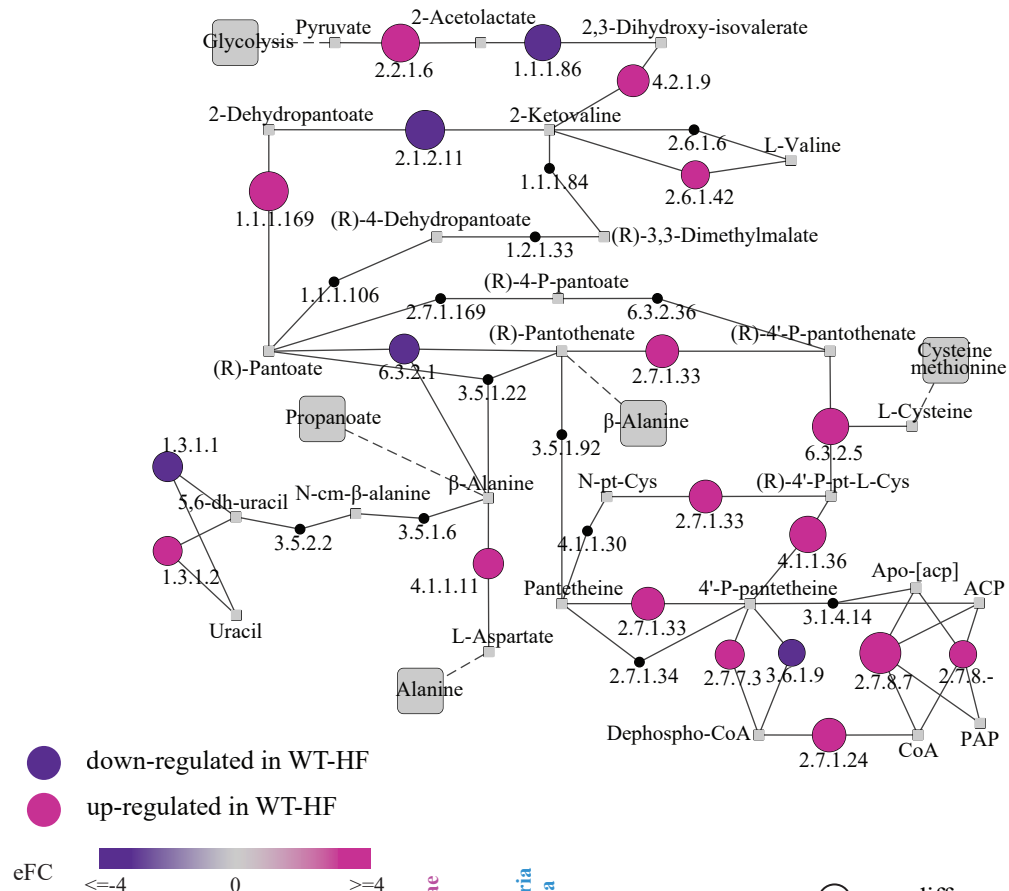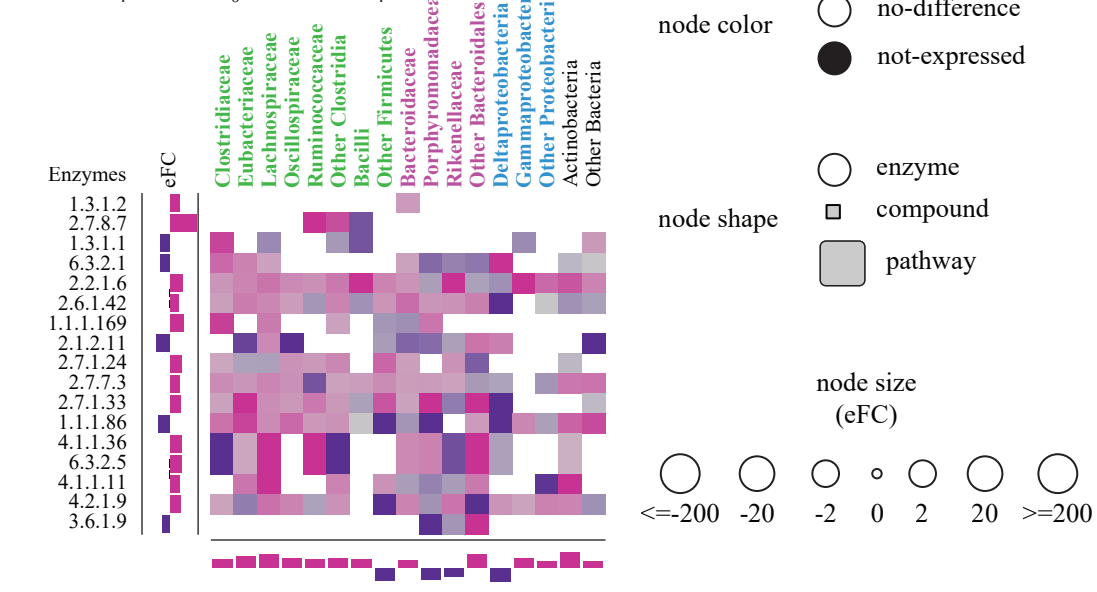

Supplement: Supplementary file 10 — Diet-based comparisons of enzyme expression in pantothenate pathway. Two comparisons are shown: (A) Plin2-HF vs. Plin2-LF and (B) WT-HF vs. WT-LF. Circular nodes indicate enzymes, with size indicating relative difference in expression between sample types and color indicating direction of change (see inset key). Associated heatmaps indicate global changes in expression for each enzyme, in addition to taxon-specific changes in expression for each of the 17 defined taxa colored according to phylum. The following abbreviations are used: 5,6-dh-uracil (5,6-dihydro-uracil), N-cm-β-alanine (N-carbamoyl-β-alanine), N-pt-Cys (N-pantothenoyl-cysteine), and (R)-4′-P-pt-L-Cys ((R)-4′-phospho-pantothenoyl-l-cysteine. (PDF 1119 kb) [file 40168_2017_327_MOESM10_ESM.pdf]

(A) Species recovered

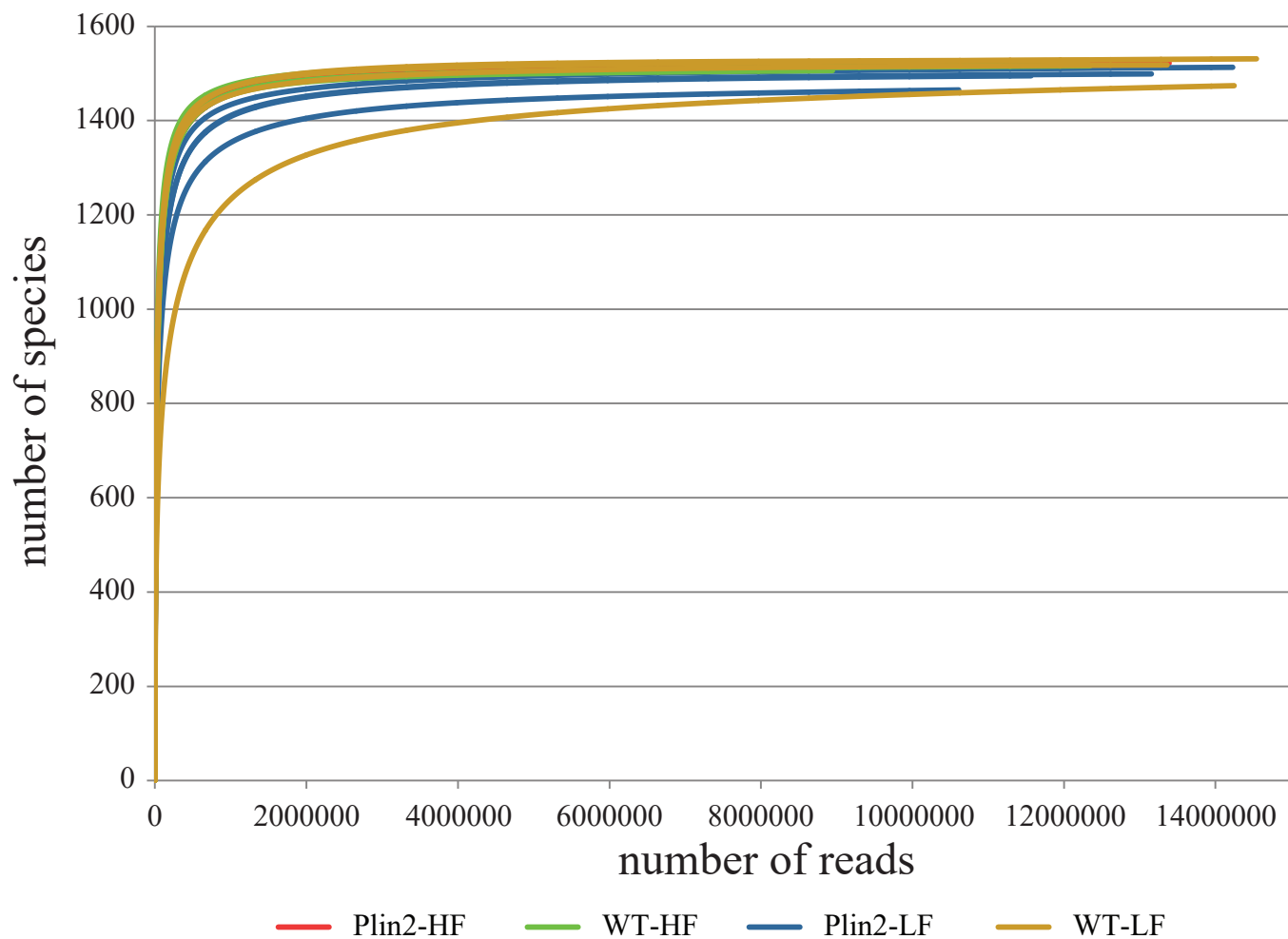

(B) Enzymes recovered

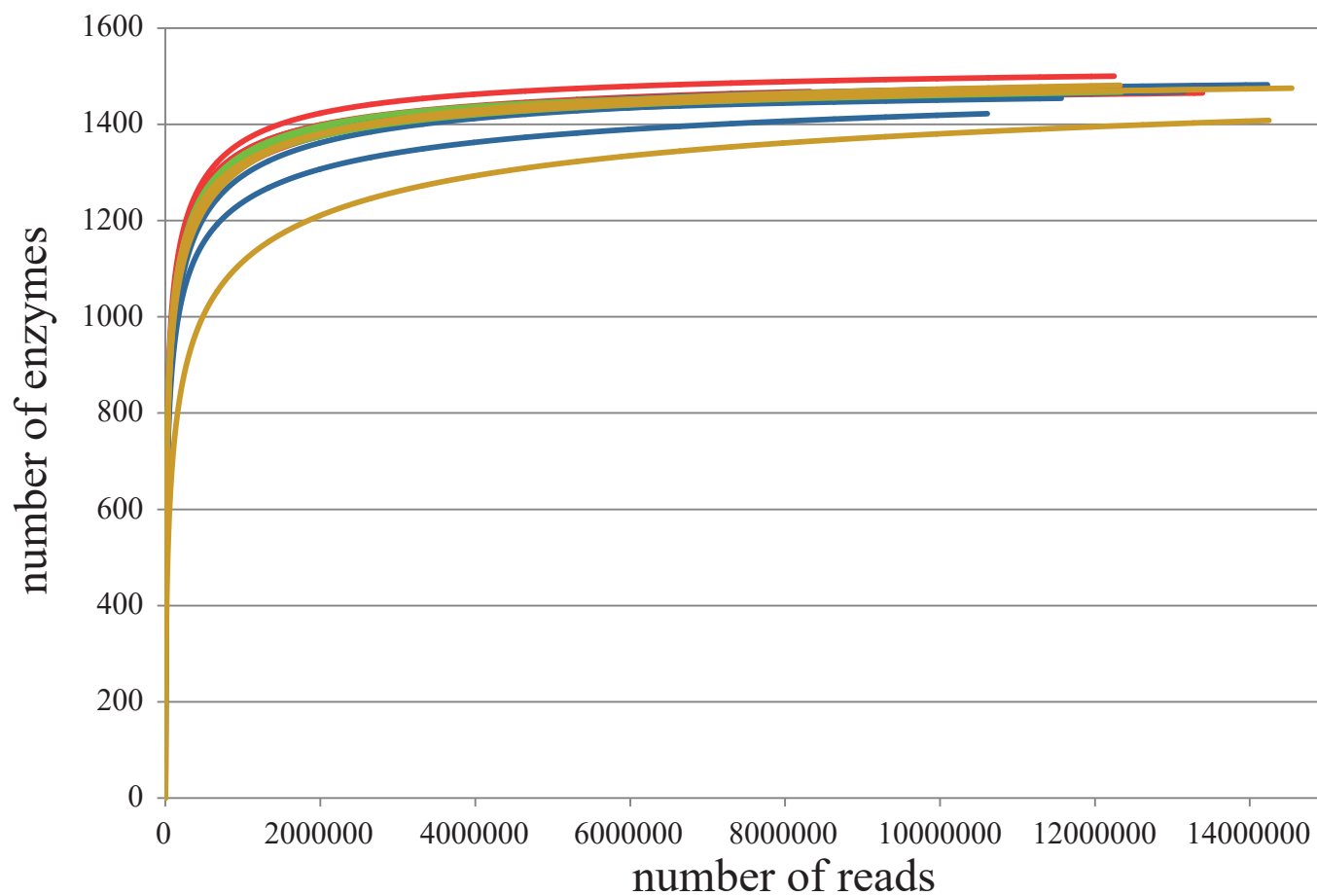

Supplement: Supplementary file 14 — Rarefaction analysis of annotated mRNA reads. Recovery of species (A) and enzymes (B) with increasing numbers of annotated mRNA reads (reads mapped to known transcripts) indicate that sequencing depth for each sample was sufficient to recover the vast majority of species and enzymes present within each of the 16 samples. Rarefaction analysis was performed using R. (PDF 16825 kb) [file 40168_2017_327_MOESM14_ESM.pdf]
