# Supplementary material for: Perilipin-2 modulates dietary fat-induced microbial global gene expression profiles in the mouse intestine
Source: Microbiome. 2017 Sep 6;5:117. doi: 10.1186/s40168-017-0327-x (PMC5588750; doi:10.1186/s40168-017-0327-x)
Supplement: Supplementary file 9 — Genotype-based comparisons of pantothenate pathway enzyme expression. Two comparisons are shown: (A) Plin2-HF vs. WT-HF and (B) Plin2-LF vs. WT-LF. Circular nodes indicate enzymes, with size indicating relative difference in expression between sample types and color indicating direction of change (see inset key). Associated heatmaps indicate global changes in expression for each enzyme, in addition to taxon-specific changes in expression for each of the 17 defined taxa colored according to phylum. The following abbreviations are used: 5,6-dh-uracil (5,6-dihydro-uracil), N-cm-β-alanine (N-carbamoyl-β-alanine), N-pt-Cys (N-pantothenoyl-cysteine), and (R)-4′-P-pt-L-Cys ((R)-4′-phospho-pantothenoyl-l-cysteine. (PDF 1370 kb) [file 40168_2017_327_MOESM9_ESM.pdf]

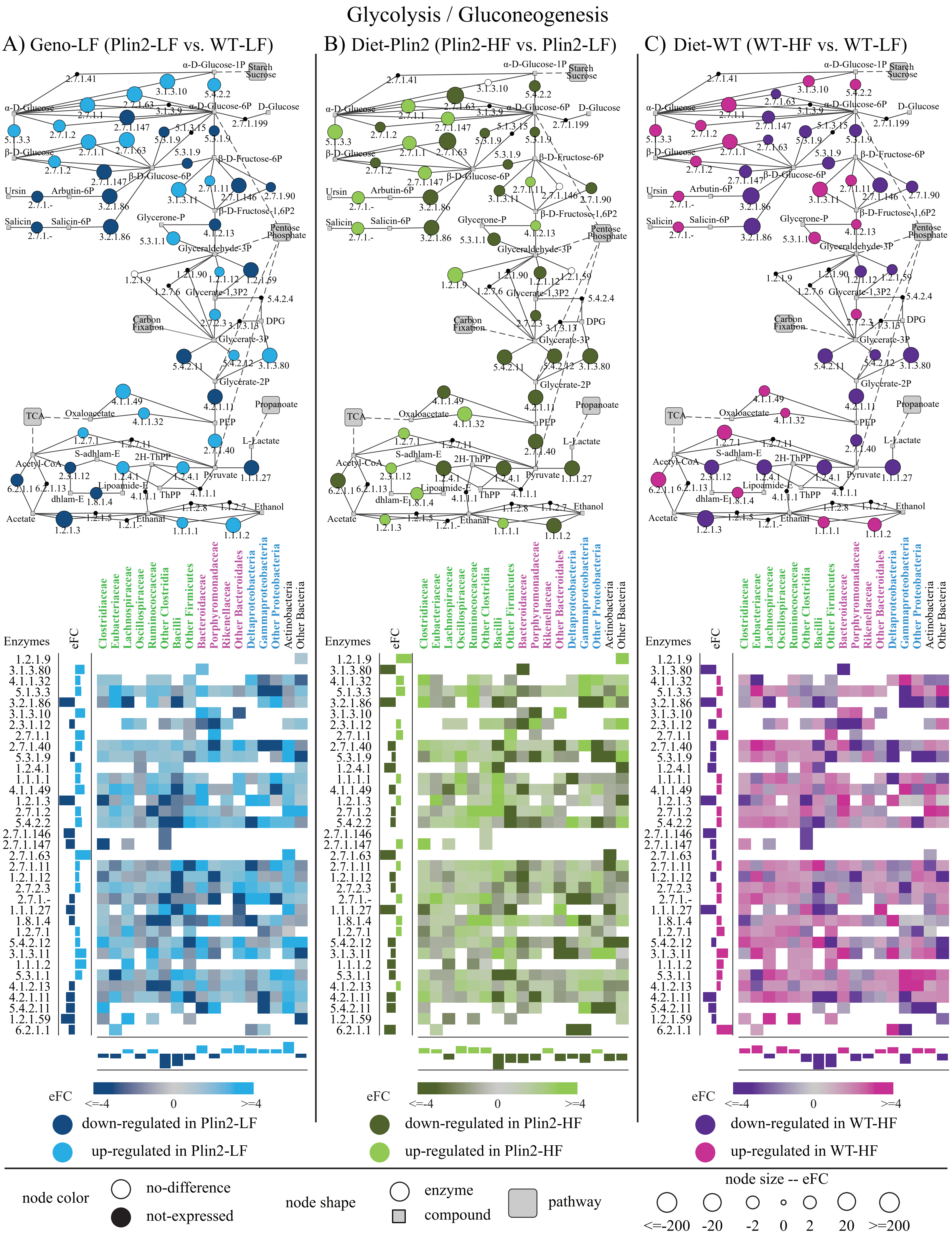
Glycolysis / Gluconeogenesis
A) Geno-LF (Plin2-LF vs. WT-LF)
B) Diet-Plin2 (Plin2-HF vs. Plin2-LF)
C) Diet-WT (WT-HF vs. WT-LF)
α-D-Glucose-1P
Starch Sucrose
α-D-Glucose
α-D-Glucose-6P
D-Glucose
β-D-Glucose
β-D-Glucose-6P
β-D-Fructose-6P
β-D-Fructose-1,6P2
Ursin
Arbutin-6P
Salicin
Salicin-6P
Glycerone-P
Pentose Phosphate
Glyceraldehyde-3P
Glycerate-1,3P2
Carbon Fixation
DPG
Glycerate-3P
Glycerate-2P
Propanoate
TCA
Oxaloacetate
PEP
L-Lactate
S-adhlam-E
2H-ThPP
Acetyl-CoA
Pyruvate
Lipoamide-E
dhlam-E
ThPP
Ethanol
Acetate
Ethanal
Enzymes
eFC
Clostridiaceae
Eubacteriaceae
Lachnospiraceae
Oscillospiraceae
Ruminococcaceae
Other Clostridia
Bacilli
Other Firmicutes
Bacteroidaceae
Porphyromonadaceae
Rikenellaceae
Other Bacteroidales
Deltaproteobacteria
Gammaproteobacteria
Other Proteobacteria
Actinobacteria
Other Bacteria
1.2.1.9
3.1.3.80
4.1.1.32
5.1.3.3
3.2.1.86
3.1.3.10
2.3.1.12
2.7.1.1
2.7.1.40
5.3.1.9
1.2.4.1
1.1.1.1
4.1.1.49
1.2.1.3
2.7.1.2
5.4.2.2
2.7.1.146
2.7.1.147
2.7.1.63
2.7.1.11
1.2.1.12
2.7.2.3
2.7.1.-
1.1.1.27
1.8.1.4
1.2.7.1
5.4.2.12
3.1.3.11
1.1.1.2
5.3.1.1
4.1.2.13
4.2.1.11
5.4.2.11
1.2.1.59
6.2.1.1
eFC
<=-4
0
>=4
down-regulated in Plin2-LF
up-regulated in Plin2-LF
down-regulated in Plin2-HF
up-regulated in Plin2-HF
down-regulated in WT-HF
up-regulated in WT-HF
node color
no-difference
not-expressed
node shape
enzyme
compound
pathway
node size -- eFC
<=-200
-20
-2
0
2
20
>=200
