# Supplementary material for: Perilipin-2 modulates dietary fat-induced microbial global gene expression profiles in the mouse intestine
Source: Microbiome. 2017 Sep 6;5:117. doi: 10.1186/s40168-017-0327-x (PMC5588750; doi:10.1186/s40168-017-0327-x)
Supplement: Supplementary file 11 — Genotype-based comparisons of enzyme expression in butanoate pathway. Two comparisons are shown: (A) Plin2-HF vs. WT-HF and (B) Plin2-LF vs. WT-LF. Circular nodes indicate enzymes, with size indicating relative difference in expression between sample types and color indicating direction of change (see inset key). Associated heatmaps indicate global changes in expression for each enzyme, in addition to taxon-specific changes in expression for each of the 17 defined taxa colored according to phylum. The following abbreviations are used: 3B–CoA (3-butenoyl-CoA), 4HB-CoA (4-hydroxy-butanoyl-CoA), C-CoA (crotonoyl-CoA), G-CoA (glutaconyl-CoA), 3HB-CoA (3-hydroxybutanoyl-CoA), and HMG-CoA (hydroxy-3-methylglutaryl-CoA). (PDF 1234 kb) [file 40168_2017_327_MOESM11_ESM.pdf]

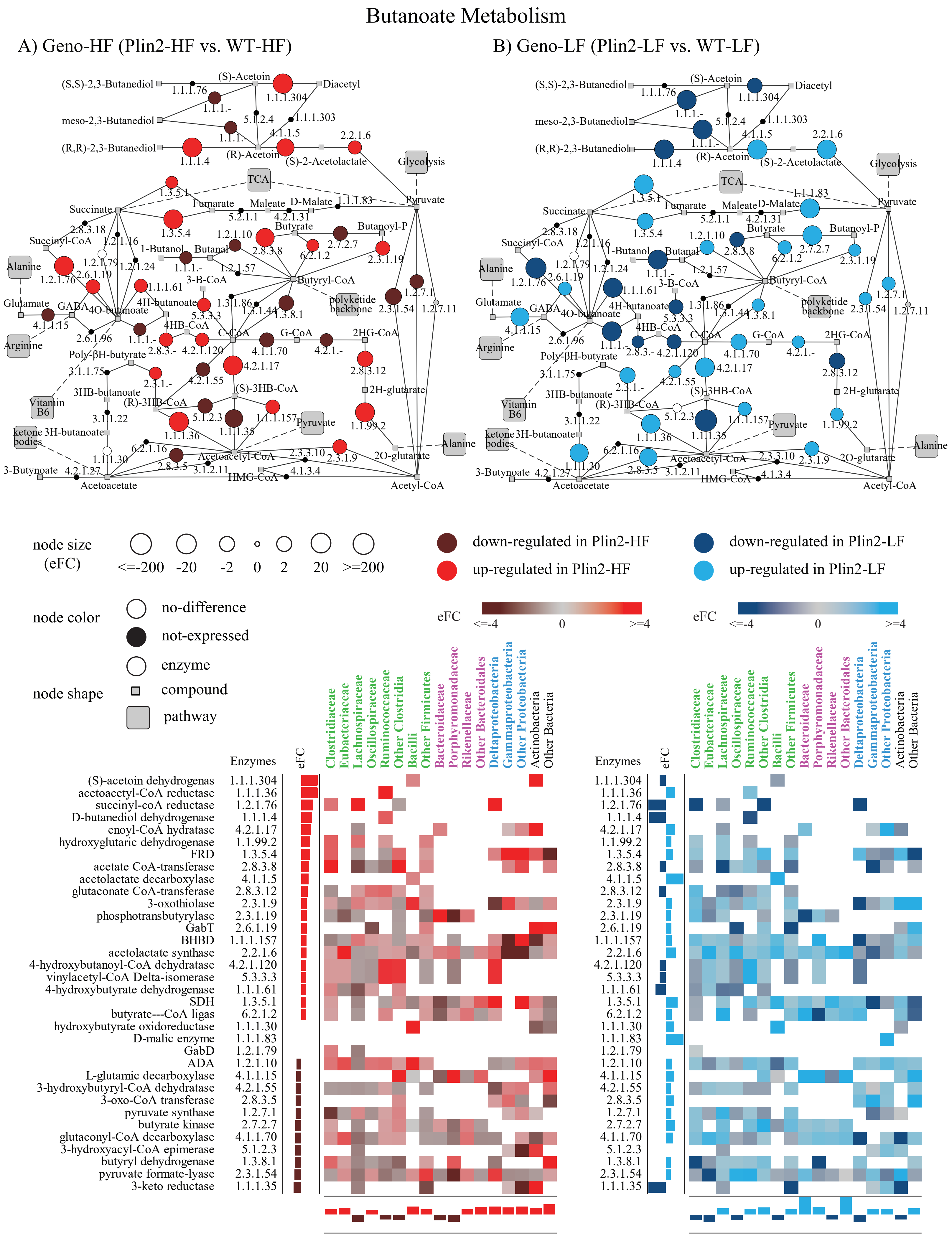
Butanoate Metabolism
A) Geno-HF (Plin2-HF vs. WT-HF)
B) Geno-LF (Plin2-LF vs. WT-LF)
node size (eFC)
<=-200 -20 -2 0 2 20 >=200
down-regulated in Plin2-HF
up-regulated in Plin2-HF
down-regulated in Plin2-LF
up-regulated in Plin2-LF
node color
no-difference
not-expressed
enzyme
node shape
compound
pathway
eFC
<=-4 0 >=4
Enzymes
Clostridiaceae
Eubacteriaceae
Lachnospiraceae
Oscillospiraceae
Ruminococcaceae
Other Clostridia
Bacilli
Other Firmicutes
Bacteroidaceae
Porphyromonadaceae
Rikenellaceae
Other Bacteroidales
Deltaproteobacteria
Gammaproteobacteria
Other Proteobacteria
Actinobacteria
Other Bacteria
(S)-acetoin dehydrogenas 1.1.1.304
acetoacetyl-CoA reductase 1.1.1.36
succinyl-coA reductase 1.2.1.76
D-butanediol dehydrogenase 1.1.1.4
enoyl-CoA hydratase 4.2.1.17
hydroxyglutaric dehydrogenase 1.1.99.2
FRD 1.3.5.4
acetate CoA-transferase 2.8.3.8
acetolactate decarboxylase 4.1.1.5
glutaconate CoA-transferase 2.8.3.12
3-oxothiolase 2.3.1.9
phosphotransbutyrylase 2.3.1.19
GabT 2.6.1.19
BHBD 1.1.1.157
acetolactate synthase 2.2.1.6
4-hydroxybutanoyl-CoA dehydratase 4.2.1.120
vinylacetyl-CoA Delta-isomerase 5.3.3.3
4-hydroxybutyrate dehydrogenase 1.1.1.61
SDH 1.3.5.1
butyrate---CoA ligas 6.2.1.2
hydroxybutyrate oxidoreductase 1.1.1.30
D-malic enzyme 1.1.1.83
GabD 1.2.1.79
ADA 1.2.1.10
L-glutamic decarboxylase 4.1.1.15
3-hydroxybutyryl-CoA dehydratase 4.2.1.55
3-oxo-CoA transferase 2.8.3.5
pyruvate synthase 1.2.7.1
butyrate kinase 2.7.2.7
glutaconyl-CoA decarboxylase 4.1.1.70
3-hydroxyacyl-CoA epimerase 5.1.2.3
butyryl dehydrogenase 1.3.8.1
pyruvate formate-lyase 2.3.1.54
3-keto reductase 1.1.1.35
