# Supplementary material for: Perilipin-2 modulates dietary fat-induced microbial global gene expression profiles in the mouse intestine
Source: Microbiome. 2017 Sep 6;5:117. doi: 10.1186/s40168-017-0327-x (PMC5588750; doi:10.1186/s40168-017-0327-x)

Butanoate Metabolism

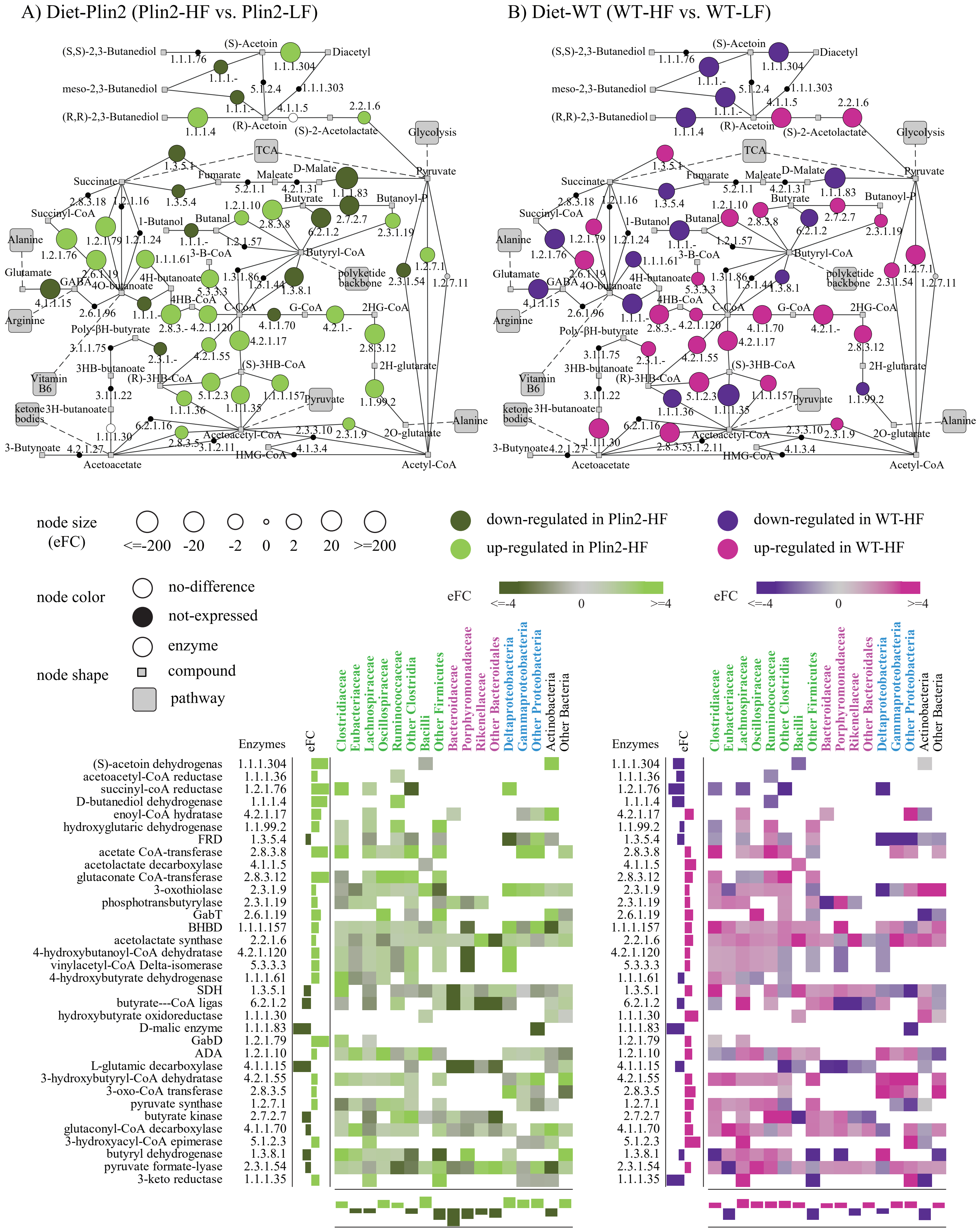

Supplement: Supplementary file 12 — Diet-based comparisons of enzyme expression in butanoate pathway. Two comparisons are shown: (A) Plin2-HF vs. Plin2-LF and (B) WT-HF vs. WT-LF. Circular nodes indicate enzymes, with size indicating relative difference in expression between sample types and color indicating direction of change (see inset key). Associated heatmaps indicate global changes in expression for each enzyme, in addition to taxon-specific changes in expression for each of the 17 defined taxa colored according to phylum. The following abbreviations are used: 3B–CoA (3-butenoyl-CoA), 4HB-CoA (4-hydroxy-butanoyl-CoA), C-CoA (crotonoyl-CoA), G-CoA (glutaconyl-CoA), 3HB-CoA (3-hydroxybutanoyl-CoA), HMG-CoA (hydroxy-3-methylglutaryl-CoA). (PDF 1262 kb) [file 40168_2017_327_MOESM12_ESM.pdf]
